# AI-Driven Secondary Immunomodulatory Effects of Conventional Drugs on Patient-Derived Macrophages

**DOI:** 10.3390/ijms27093894

**Published:** 2026-04-27

**Authors:** Igor D. Zlotnikov, Alexander A. Vinogradov, Elena V. Kudryashova

**Affiliations:** 1 Laboratory of Targeted Therapy and Differential Diagnosis, Central University, 7 Gasheka St., Moscow 123056, Russia; izlotnikov2003@yandex.ru; 2 Faculty of Chemistry, Lomonosov Moscow State University, Leninskie Gory, 1/3, Moscow 119991, Russia; 3 Department of Mathematics, Centre for Mathematical Plasma Astrophysics, KU Leuven, 3001 Leuven, Belgium; alexander.vinogradov@kuleuven.be

**Keywords:** macrophage polarization, bronchoalveolar lavage fluid (BALF), personalized medicine, M1/M2 macrophages, antibiotics, cytostatic drug, in vitro remodeling, AI, Linear Discriminant Analysis

## Abstract

The secondary immunomodulatory effects of conventional therapeutics, such as antibiotics and cytostatics, are frequently overlooked despite their significant clinical implications. Building on our previous findings that drugs like paclitaxel and doxorubicin heavily influence macrophage polarization—potentially driving metastasis or inflammation—this study systematically evaluates the secondary immune-modulating actions of standard drugs and natural adjuvants. Using patient-derived bronchoalveolar lavage (BAL) fluid (ex vivo alveolar macrophages), we developed an analytical platform using synthetic carbohydrate-functionalized fluorescent ligands targeting key receptors (CD206, CD209, CD280, CD301). Integrating ligand-binding profiles with Linear Discriminant Analysis (LDA) yielded quantitative immune-state vectors capable of differentiating favorable and unfavorable prognostic signatures and imbalanced immune states. Pro-filing samples across heterogeneous respiratory conditions revealed highly con-text-dependent responses. While some treatments synergistically corrected unfavorable imbalanced profiles, others provoked dysregulation. Notably, in pneumonia or bronchitis with an asthma-prone M2-dominant profile, specific antibiotic regimens are critical; doxycycline, for instance, may exacerbate patient deterioration by further driving M2a polarization. Crucially, we identified that natural adjuvants (e.g., curcumin, coumarins, polyphenols) exhibit potent properties capable of correcting these adverse secondary drug effects. Ultimately, this profiling platform highlights the necessity of evaluating patient-specific secondary drug effects, offering a functional blueprint for precision immunotherapy and the rational design of adjuvant-enhanced treatments.

## 1. Introduction

Macrophages are highly dynamic and functionally heterogeneous populations of innate immune cells that play multifaceted roles in tissue homeostasis, immune surveillance, and host defense [1]. These cells are distributed throughout virtually all tissues, where they function as central regulators of the immune response [2]. A defining characteristic of macrophages is their exceptional functional plasticity—their capacity to rapidly adapt phenotype and function in response to diverse microenvironmental signals, including microbial products, damaged cells, and specific cytokines [3,4,5,6,7]. This adaptability positions macrophages as key players in both the successful resolution of inflammation and the development of chronic pathologies.

The classical M1/M2 polarization paradigm provides a fundamental framework for understanding macrophage function, categorizing them into two functionally distinct subsets [8,9,10,11,12,13]. M1, or classically activated, macrophages are induced by pro-inflammatory signals such as interferon-gamma (IFN-γ) and lipopolysaccharide (LPS). They are characterized by production of pro-inflammatory cytokines (TNF-α, IL-6, IL-12), possess potent microbicidal and cytotoxic properties, and are crucial for mounting Th1-type immune responses against intracellular pathogens and tumors [14,15,16]. In contrast, M2, or alternatively activated, macrophages are induced by anti-inflammatory cytokines (IL-4, IL-13) and are involved in immunoregulation, wound healing, tissue remodeling, and angiogenesis, primarily through secretion of anti-inflammatory agents such as IL-10 [17,18,19,20,21].

However, the binary M1/M2 model is now recognized as an oversimplification. In vivo, macrophage phenotypes exist not as discrete states but as a dynamic continuum, with intermediate and hybrid profiles shaped by the complex local tissue environment [22,23,24]. For instance, recent studies (Sun et al., 2026) revealed that distinct tissue niches drive divergent immune responses: unlike liver injuries dominated by lymphocytes, cardiac trauma recruits specific CCRL2+ macrophages that co-express pro-inflammatory cytokines yet primarily drive fibrosis through direct fibroblast modulation [25]. Crucially, dysregulation of macrophage polarization axis is a defining feature of numerous pathologies, where the dominant macrophage profile often dictates disease progression and clinical outcome [26].

In oncology, prevalence of M2-like tumor-associated macrophages (TAMs) is well-documented as a factor for poor prognosis, promoting tumor growth, angiogenesis, metastasis, and multidrug resistance [8,27,28,29,30]. In tuberculosis infection, M1 macrophage activation is essential for controlling *Mycobacterium tuberculosis* [31,32], while insufficient M1 activity fails to eliminate the pathogen, leading to chronic infection [33,34]. Similarly, in pulmonary diseases such as idiopathic pulmonary fibrosis (IPF) and chronic obstructive pulmonary disease (COPD), persistent aberrant macrophage activation within the bronchoalveolar lavage fluid is associated with disease severity and unresolved inflammation [35,36]. Although recent comprehensive and methodologically complex efforts have highlighted that clinical trajectories are dictated by specific functional macrophage ‘fingerprints’ rather than a binary M1/M2 ratio [37], these massive studies have thus far provided limited practical understanding or translational value.

Recent evidence, including our own work on doxorubicin-induced macrophage modulation [38], revealed that the immunomodulatory impact of drugs constitutes a critical “hidden layer” of pharmacology [39]. While primarily designed to target malignancies or pathogens, many pharmacological agents—such as cytostatics and antibiotics—exhibit secondary activities that inadvertently remodel the immune landscape. These off-target effects can profoundly influence macrophage polarization, creating a therapeutic paradox. For example, despite its potent cytostatic efficacy, we recently demonstrated that doxorubicin can paradoxically induce an immunosuppressive M2-like macrophage phenotype [38]. This shift fosters a pro-tumorigenic environment associated with the emergence of tumor-associated macrophages (TAMs), promoting metastatic dissemination and contributing to multidrug resistance [40].

Conversely, other agents can restore antitumor immunity. Paclitaxel, a classical microtubule-stabilizing chemotherapeutic, promotes a pro-inflammatory M1 phenotype by activating Toll-like receptor 4 (TLR4) signaling and amplifying NF-κB–mediated transcriptional programs [41,42], effectively converting immunologically “cold” (M2-rich) tumors into “hot” immune-active niches.

Importantly, the context-dependent nature of these phenotypic transitions underscores that macrophage plasticity is not merely a side effect of therapy, but a primary therapeutic target that can be harnessed to optimize treatment outcomes. To map these complex dynamics, it is crucial to consider a diverse array of pharmacological agents spanning multiple therapeutic classes, including antibiotics, cytostatics, and adjuvants such as terpenoid-based efflux pump inhibitors, metabolic regulators, and microenvironment modifiers.

Among these, curcuminoids—a bioactive compounds renowned for its potent anti-inflammatory activity via NF-κB inhibition—also act as an effective TAM reprogrammer in cancer contexts [43,44]. Given its previously demonstrated direct synergistic cytotoxicity when combined with doxorubicin against cancer cells [45,46], curcuminoids emerge as perspective adjuvant candidates. Similarly, coumarins and their derivatives, such as 4-methylumbelliferone (MUmb), exhibit intricate, context-dependent immunomodulatory properties [47,48], positioning them as promising adjuvants for chemotherapy.

The landscape of potential immunomodulators extends further to agents with highly nuanced immune profiles. For instance, the broad-spectrum antibiotic doxycycline, while classically defined as an anti-inflammatory agent, exerts secondary effects as an inhibitor of matrix metalloproteinases, and emerging evidence indicates it may also actively inhibit M2 polarization [49,50]. Other notable modulators include the antioxidant glutathione [10], which influences macrophage polarization via oxidative stress pathways, and the antidiabetic agent metformin, known to attenuate M2 polarization through the activation of the AMPK (AMP activated protein kinase) pathway. Beyond these established therapeutics, novel synthetic compounds—specifically chromene derivatives [51] and chalcone analogs [52]—offer profound potential to actively drive macrophage repolarization.

The ex vivo platform presented in this study offers a robust and highly relevant system for screening novel synthetic drugs, nutraceuticals, and immunoadjuvants. Ultimately, rational drug design must integrate these complex immunomodulatory dynamics to accurately predict the clinical outcomes. By facilitating the comprehensive assessment of macrophage repolarization prior to clinical trials, our methodology empowers precisely this predictive approach. To achieve this level of clinical relevance, the platform utilizes bronchoalveolar lavage (BAL)—a standard clinical procedure that provides a unique window into the lung’s immune landscape. Because BAL yields a rich, patient-derived cellular population comprising up to 85–98% disease-relevant alveolar macrophages, it serves as the ideal physiological model for translating these ex vivo screenings into personalized therapeutic strategies [36,53,54,55,56,57,58,59,60,61]. We have recently developed a novel methodology for high-dimensional profiling of macrophage subpopulations based on a unique panel of carbohydrate ligand markers targeting C-type lectin receptors (CD206, CD209, CD280, CD301) [37], ligand markers targeting CD68 [39], combined with assessment of phagocytic activity [62]. By applying this technique to BAL-derived macrophages, we can establish distinct functional “fingerprints” that correlate with either favorable disease resolution or poor prognosis characterized by M1/M2 imbalance.

Current approaches to macrophage phenotyping rely heavily on systemic cytokine analysis. However, these methods are largely restricted to fundamental research; despite their methodological complexity, they often yield negligible clinical utility and lack direct translational value [25]. To bridge this gap, we hypothesize that a standardized ex vivo platform applied to patient-derived alveolar macrophages constitutes a pivotal tool for personalized medicine. This approach enables the rational selection of therapies tailored to the patient’s specific immune endotype.

In this study, we establish a robust model to define prognostic polarization signatures associated with distinct clinical trajectories. By benchmarking reference pro- and anti-inflammatory agents and novel formulations, we aim to uncover latent macrophage reprogramming, thereby distinguishing therapeutic efficacy from unintended immunotoxicity. Crucially, this analytical framework facilitates the systematic evaluation of immune-adjuvants, allowing for the precise selection of optimal drug formulations. To process the complex, high-dimensional datasets generated by such evaluations, artificial intelligence approaches—including random forests and artificial neural networks—offer immense predictive potential. In the present study, we relied on Linear Discriminant Analysis (LDA) to robustly classify and model these macrophage polarization states. Consequently, this work proposes a standardized technology for the preclinical assessment of drug-induced immune remodeling, uniquely designed to predict adverse long-term effects prior to clinical application.

## 2. Results

### 2.1. Potential for Therapeutic Macrophage Reprogramming in Airway Diseases

Macrophages are pivotal orchestrators of inflammatory diseases, wielding significant control through secretion of pro-inflammatory mediators and counter-regulatory factors that drive or resolve inflammation. The remarkable plasticity of macrophages allows them to adopt diverse functional phenotypes, ranging from resting M0 state through pro-inflammatory M1 state to multiple anti-inflammatory and pro-resolution M2 subsets (M2a, M2b, M2c, M2d) [63]. Recognizing this inherent heterogeneity, we developed a comprehensive macrophage polarization profiling strategy for individual patients with distinct respiratory conditions: bronchitis (acute inflammation), bronchial asthma (allergic/eosinophilic), and bronchiectasis (chronic remodeling).

**Figure 1:**
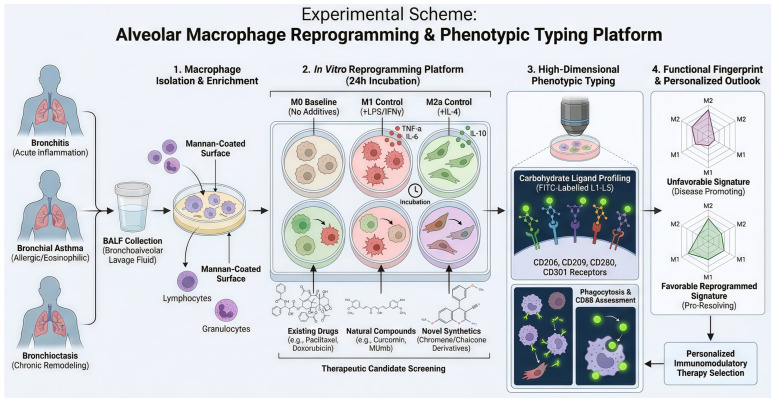
Experimental scheme for macrophage reprogramming and phenotypic typing. The platform was validated using BALF samples from patients with distinct respiratory conditions, specifically: bronchitis (acute inflammation), bronchial asthma (allergic/eosinophilic), and bronchiectasis (chronic remodeling).

Figure 1 outlines the complete workflow for isolating, treating, and phenotypically characterizing alveolar macrophages from BALF to assess therapeutic reprogramming potential. The workflow begins with isolation of alveolar macrophages from patient BALF, followed by enrichment through adherence to mannan-coated surfaces to selectively culture macrophages while excluding other immune cell types. Following isolation, macrophages are allocated to distinct experimental groups: (i) M0 baseline (no additives), (ii) M1 polarization control (LPS + IFNγ), (iii) M2a polarization control (IL-4), and experimental groups treated with pharmacological agents. Following a 24 h period of incubation, macrophages are subjected to phenotypic profiling using a panel comprising five FITC-labeled carbohydrate ligands, designated L1 through L5, each bearing mannose, galactose, or trimannose fragments that target specific receptors such as CD206, CD209, CD280, and CD301. This multiparametric approach enables precise discrimination of M0, M1, and M2a phenotypes based on unique receptor expression patterns, quantified through fluorescence intensity and ligand binding analysis.

The resultant polarization profiles allow objective tracking of therapeutic remodeling and identification of compounds that effectively shift macrophage populations between M1 and M2 states, or toward balanced M0/M1/M2 ratios conducive to disease resolution. Expected outcomes include discovery of novel therapeutic agents and deeper mechanistic understanding of their actions at the carbohydrate receptor level, paving the way for personalized immunomodulatory strategies to combat severe respiratory diseases.

### 2.2. Development and Characterization of Carbohydrate-Functionalized Fluorescent Ligands for Multiparametric Macrophage Typing

#### 2.2.1. Physicochemical Parameters and Design Rationale

In order to conduct a comprehensive analysis of alveolar macrophages subpolulations profile, we implemented a series of fluorescent ligands based on carbohydrates (Figure S1). Based on our previous research [64], which involved the synthesis of FITC-labeled polymers conjugated with specific carbohydrate units, here the ligands panel refined to selectively bind to diverse macrophage surface receptors and ensure precise phenotypic discrimination. The ligands were designed based on systematic variation in carbohydrate type (mannose vs. galactose), configuration (linear vs. cyclic), and degree of functionalization to optimize receptor binding specificity and efficiency for discriminating M0, M1, M2a, M2b, M2c, and M2d macrophage subpopulations.

Figure S1 provides a schematic overview of the synthetic approach employed to generate these FITC-labeled fluorescent probes, incorporating five distinct carbohydrates with receptor-specific recognition properties. Successful synthesis of the fluorescently labeled polymeric conjugates is corroborated by spectroscopic analyses. FTIR spectra (Figure S2) clearly show characteristic vibrational modes of PEI backbone (CH2 stretching at 2980–2800 cm^−1^) and FITC label (C=C at 1580 cm^−1^ and 1450 cm^−1^), along with C–N and C–O–C bonds indicative of PEI-saccharide conjugation (1200–1000 cm^−1^). ^1^H NMR data further confirms the successful assembly (Figure S3), displaying expected proton signals for the appended carbohydrate moieties (e.g., mannose signals at 3.9–5.3 ppm) alongside characteristic PEI polymer resonances (around 2.5–2.9 ppm). The absence of anomeric proton signals in some Gal-PEI and Man-PEI conjugates, along with slight shifts in carbohydrate proton signals, suggests successful modification and conjugation at the reducing end of the sugar residues. Table 1 summarizes the designation, physicochemical parameters, and specificity of carbohydrate-functionalized fluorescent ligands for macrophage receptors.

To mechanistically validate receptor specificity and precisely delineate the cellular polarization status, we employed competitive inhibition assays with mannan. As a polymannose polysaccharide, mannan acts as a specific competitive inhibitor for the carbohydrate recognition domains of CD206 and CD209, allowing us to establish distinct functional profiles for each macrophage subpopulation. The identification of classically activated M1 macrophages inherently relies on the lower degree of mannan inhibition. Because the M1 phenotype characterized by lower expression of mannose-specific receptors, the binding of fluorescent ligands persists unabated even in the presence of the inhibitor. Conversely, the M2a phenotype, representing the prototypical alternatively activated state, exhibits robust expression across the entire targeted receptor panel—including CD206, CD209, CD280, and CD301. Consequently, M2a cells display maximal initial multivalent ligand binding, which is subsequently and profoundly abrogated following mannan competition. The regulatory M2b subtype, in contrast, is characterized by a weak, residual expression of CD206 and a absence of CD209 and CD301, resulting in an overall low binding profile with minimal susceptibility to mannan blockade. Furthermore, M2c macrophages, typically associated with tissue remodeling, present high CD206 and moderate CD280 levels but lack CD209 and CD301; thus, they exhibit strong mannan-inhibited binding of mannose-based ligands while remaining unresponsive to galactose-targeted probes, distinctly separating them from the M2a cluster. Finally, unpolarized M0 macrophages maintain a low expression of mannose receptors without definitive activation markers.

#### 2.2.2. Flow Cytometry Analysis of Ligand Binding Specificity in Primary Ex Vivo BALF Macrophages and Standardized Cellular Controls (THP-1 Macrophages)

Prior to evaluating the specific receptor-targeting efficacy of the synthesized polymer-ligand conjugates in complex clinical samples, flow cytometry was employed to establish fundamental binding profiles and confirm receptor specificity within a controlled in vitro setting. We rigorously ascertained the overall cellularity and viability across standardized, uniformly polarized THP-1 macrophages. As illustrated in Figure S4a (Supplementary Materials), the engineered ligands exhibited distinct, structure-dependent binding affinities in this purified CD206+ cellular model.

The branched trimannose (L5, triMan-GlcNAc2-PEI-FITC) and cyclic mannose (L2, ManCyc-PEI-FITC) displayed pronounced rightward shifts in histograms, with mean fluorescence intensity (MFI) values 3–4-fold higher than the control. Specifically, the cyclic derivative L2 demonstrated significantly higher binding efficiency (~60%) compared to the linear analogue L1 (ManLin-PEI-FITC, ~38%), suggesting that the cyclic conformation provides an optimized geometry for C-type lectin receptors such as CD206. A similar trend was observed for galactose-targeting ligands, where the cyclic scaffold L4 (GalCyc-PEI-FITC) significantly outperformed its linear counterpart L3 (GalLin-PEI-FITC) (~48% vs. ~14%), confirming the structural advantage of cyclic systems for lectin recognition in a homogeneous environment. Importantly, the overall binding efficiency of the galactose-based ligands was substantially lower than that of their mannosylated counterparts. This distinct preference for mannose over galactose confirms the successful differentiation of the THP-1 model into a robust, CD206-rich (mannose receptor-positive) macrophage population, demonstrating that the binding is driven by specific receptor avidity rather than indiscriminate carbohydrate uptake.

To further verify that this pronounced cellular interaction is mediated by specific receptor-ligand recognition, CD206-negative human fibroblasts were employed as a negative control (Figure S4). While the highly active branched construct (triMan-GlcNAc_2_-PEI-FITC) induced a massive ~80% binding shift in CD206+ macrophages, it yielded only a minimal baseline shift (17.4%) in the fibroblast population, with the linear analogue (ManLin-PEI-FITC) demonstrating a near-complete absence of binding (4.25%). This stark contrast definitively validates the high receptor-mediated specificity of the carbohydrate-functionalized platform.

**Figure 2:**
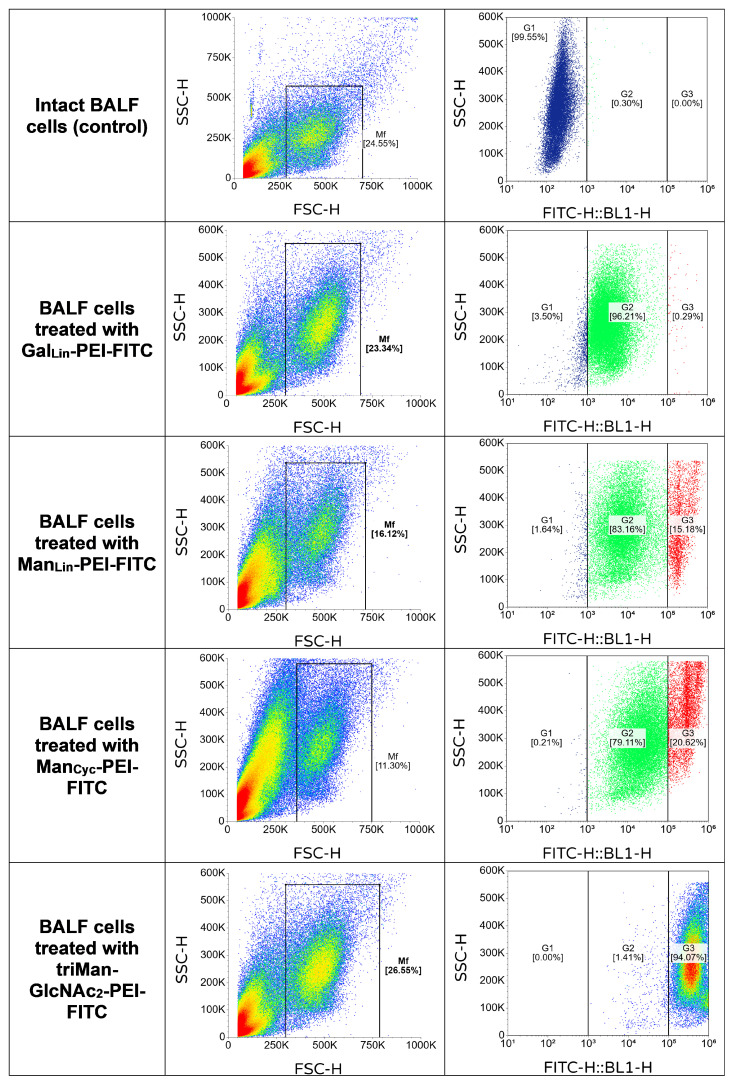
Flow cytometric analysis of specific ligand binding profiles in patient-derived BALF macrophages. Representative dot plots (Left: SSC-H vs. FITC-H) and corresponding fluorescence intensity histograms (Right: Count vs. FITC-H) are shown for each condition. To accurately evaluate the affinity gradient of the synthesized panel, a strict three-gate strategy was uniformly applied across all samples: G1 indicates unbound cells; G2 indicates moderate surface interaction; and G3 indicates high-intensity, high-affinity receptor binding. Intact BAL cells (negative control), localized predominantly in G1 (99.65%). Cells incubated with linear FITC-PEI-galactose (low-affinity reference), demonstrating significant baseline surface attachment (G2 = 96.21%) but lacking the specific affinity required for high-intensity clustering (G3 = 0.29%). Cells incubated with linear FITC-PEI-mannose, showing an affinity-driven shift into the specific high-binding G3 gate (15.18%). Cells incubated with cyclic FITC-PEI-mannose, exhibiting enhanced high-affinity binding in G3 (20.63%). Cells incubated with the highly specific branched triMan-GlcNAc_2_-PEI-FITC ligand, demonstrating massive receptor engagement with an almost complete population shift directly into the high-intensity G3 gate (94.07%).

While standardized cell lines provide a crucial functional baseline and validate binding specificity, they inherently fail to capture the profound biological heterogeneity of actual clinical microenvironments. Therefore, to validate and deploy this receptor-targeting platform in a true physiological context, we advanced our analysis to primary alveolar macrophages isolated directly from patient BALF (Figure 2).

To accurately capture the heterogeneity of these primary samples and distinctly differentiate moderate baseline interactions from high-affinity receptor engagement, a rigorous three-gate strategy was employed: G1 representing the unbound cell population, G2 representing cells with moderate surface interaction, and G3 representing a subpopulation with high-intensity, high-affinity ligand binding. The baseline threshold was established using intact BAL cells (negative control, Figure 2), where the vast majority of events localized in the unbound G1 gate (99.65%), with negligible background in G2 (0.30%) and G3 (0%).

The application of this gating strategy revealed a fascinating, two-step dynamic of ligand-cell interactions. The linear galactose ligand (GalLin-PEI-FITC, L3), acting as a low-affinity reference, successfully initiated cellular interaction, drastically depleting the unbound G1 pool (down to 3.50%). However, the population almost entirely arrested within the moderate G2 gate (96.21%), with a negligible presence in the highly positive G3 gate (0.29%, Figure 2). This confirms that while the polymer backbone facilitates basal surface attachment, the low-affinity galactose construct fails to trigger the profound, specific receptor clustering required to cross the high-intensity G3 threshold.

In stark contrast, mannosylated ligands demonstrated a progressive, affinity-driven capacity to overcome the G2 barrier and drive specific receptor clustering. The linear mannose ligand (ManLin-PEI-FITC) and cyclic mannose derivative (ManCyc-PEI-FITC) successfully transitioned substantial fractions of the clinical macrophage population into the high-binding G3 zone, capturing 15.18% (Figure 2) and 20.63% (Figure 2), respectively. Crucially, the fundamental structure-activity relationship—where the cyclic conformation outperforms the linear analogue—remained perfectly conserved between the in vitro model and the ex vivo clinical reality.

Most remarkably, the complex branched trimannose ligand (triMan-GlcNAc2-PEI-FITC, L5) completely overcame the clinical heterogeneity of the BALF samples. It virtually depleted the G1 pool (0%) and almost completely bypassed the moderate G2 pool (1.41%), shifting an astonishing 94.07% of the entire primary macrophage population directly into the high-intensity G3 gate (Figure 2).

Statistical analysis of the flow cytometry data across the cohort using a one-way analysis of variance (ANOVA) followed by Tukey’s post hoc test corroborated these functional dynamics. First, all applied polymeric ligands demonstrated a statistically significant overall interaction (shift from G1 to G2/G3) compared to the intact control (*p* < 0.001). However, when isolating the high-intensity, specific receptor engagement (the G3 population), the low-affinity galactose ligand showed no significant difference from the baseline background (*p* > 0.05). Conversely, the high-intensity G3 binding of all mannose-based ligands was significantly greater than that of the galactose control (*p* < 0.05 for ManLin, *p* < 0.01 for ManCyc). Furthermore, the specific targeting efficiency of the branched triMan-GlcNAc2 ligand significantly outperformed all other monomeric constructs (*p* < 0.001), quantitatively validating its exceptional capacity to selectively cluster receptors on primary human immune cells.

#### 2.2.3. Confirmation of Carbohydrate Ligand Selectivity to Macrophage Receptors by Fluorescence Microscopy with Targeted Immunocytochemistry and Fourier-Transform Infrared (FTIR) Mapping

To ensure the reliability of the proposed in vitro profiling platform, it was crucial to confirm that the observed variations in binding affinity were driven by specific interactions between the synthesized carbohydrate ligands and their corresponding innate immune receptors on the macrophage surface. To investigate this, we applied a comprehensive analytical approach combining strict biological negative controls, fluorescence microscopy with targeted immunocytochemistry, and label-free Fourier-transform infrared (FTIR) chemical mapping.

**Figure 3:**
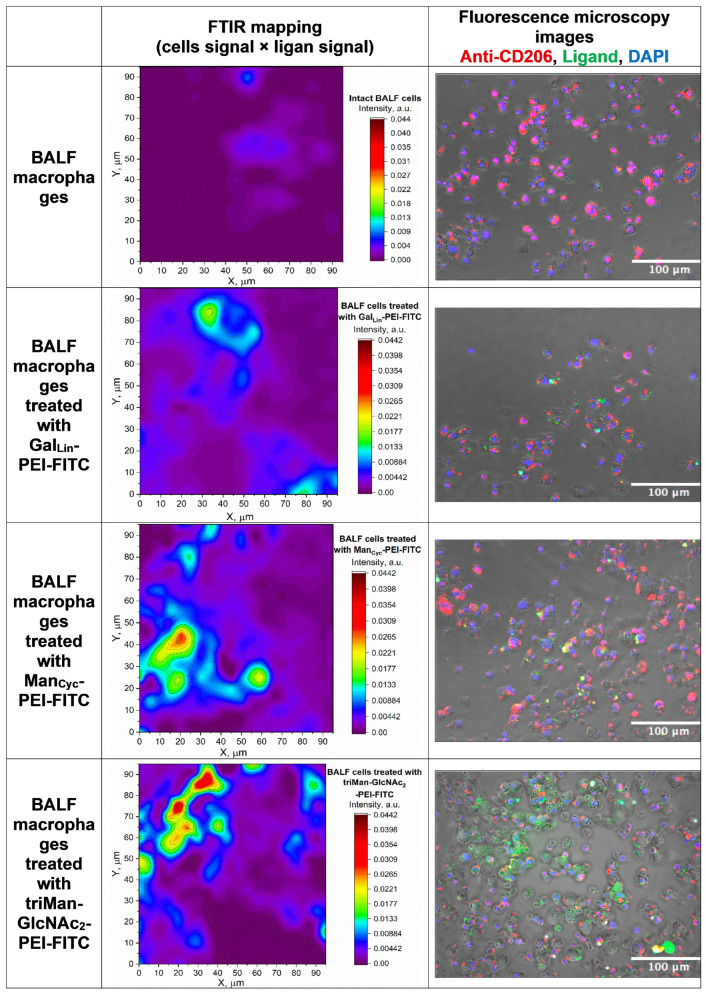
Validation of specific carbohydrate ligand binding to macrophage receptors using orthogonal imaging techniques. The left panel: label-free FTIR chemical mapping of ligand-cell interactions. The heatmaps are merge of the integral intensities of the cellular protein signal (Amide I, 1600–1700 cm^−1^) and the carbohydrate ligand signal (950–1100 cm^−1^), with warmer colors indicating higher localized intensity. The right panel: fluorescence microscopy images of BALF macrophages. Cells were stained with anti-CD206 antibodies to visualize the mannose receptor (red, ab64693, Abcam; 1:100), and nuclei were counterstained with DAPI (blue). The carbohydrate-PEI ligands are visualized via their intrinsic FITC label (green). Scale bar = 100 µm.

To visually validate this receptor-targeting specificity in the target cell population, patient-derived BALF macrophages were incubated with FITC-labeled ligands (L3, L2, and L5) and counterstained with an antibody against CD206, the canonical mannose receptor (Figure 3). Fluorescence microscopy revealed distinct, structurally dependent binding modalities. Intact (untreated) BALF cells exhibited basal CD206 expression (red fluorescence) without any interfering background signal. Following incubation with the linear galactose-based ligand (GalLin-PEI-FITC), moderate cellular binding (green) was observed; however, colocalization with the CD206 receptor was negligible. This spatial segregation aligns with the biological expectation that galactose moieties predominantly engage alternative C-type lectins, such as the macrophage galactose-type lectin (MGL/CD301), rather than the mannose receptor.

Conversely, the mannose-functionalized ligands demonstrated highly specific CD206 targeting. Macrophages treated with either ManCyc-PEI-FITC or the complex triMan-GlcNAc2-PEI-FITC exhibited intense FITC signals that strongly colocalized with the anti-CD206 focal domains, generating pronounced yellow/orange regions in the merged micrographs. This colocalization was exceptionally striking for the branched triMan-GlcNAc2 ligand, visually confirming its superior affinity for the CD206 receptor.

To rule out non-specific binding of the polyethylenimine (PEI) scaffold, CD206-negative human fibroblasts were used as a negative cellular control (Figure S4c). Microscopic analyses revealed that the mannosylated ligands (triMan-GlcNAc2-PEI-FITC) exhibited a complete absence of binding to the fibroblast population—even when administered at an extreme concentration of 10 mg/mL. This profound lack of interaction in receptor-negative cells confirms that the cellular uptake of these polymers is strictly governed by specific carbohydrate-receptor recognition.

Furthermore, to provide an independent, label-free validation at the single-cell level, we employed FTIR chemical mapping. This technique enabled the direct visualization of cellular receptor topography by tracking the distribution of the specific investigated polymer ligands. To map the specific interaction zones, we focused on extracting and overlaying two distinct spectral regions. First, the integral intensity of the Amide I band (1600–1700 cm^−1^)—which originates from the C=O stretching vibrations of peptide bonds—was used to reliably delineate the physical boundaries and the overall protein (receptor) density of the macrophage cells. Second, to trace the localization of the specific polymeric ligands, we integrated the region between 950 and 1100 cm^−1^. This specific spectral window captures the intense C–O–C stretching vibrations characteristic of the glycosidic bonds and ether linkages inherent to the carbohydrate-functionalized ligands.

By merging these two integral maps (Amide I for the cell body, and C–O–C for the ligand), we visualized the direct physical accumulation of the polymers on the cellular structures (Figure 3). This application of FTIR mapping represents a highly valuable methodological approach for single-cell analysis, offering a non-destructive confirmation of macromolecular distribution. The resulting FTIR spatial profiles directly mirrored the fluorescence topography: the highest density of carbohydrate-protein complexes—indicated by the strongest spatial overlap of the C–O–C and Amide I signals—was observed in macrophages treated with the triMan-GlcNAc2-PEI-FITC ligand. Crucially, this intense carbohydrate signature was completely absent in intact control cells and non-macrophage cells, confirming its exogenous origin from the specifically bound ligands.

The qualitative spatial data obtained from both fluorescence microscopy and our novel FTIR mapping perfectly align with the quantitative flow cytometry results and the in silico binding affinities summarized in Table 1, which are also supported by the experimental thermodynamic dissociation constants (Kdis) established in our previous work [65,67,68]. The galactose-based ligand (GalLin-PEI-FITC) showed minimal CD206 colocalization and weak localized C–O–C signal merging in FTIR maps. This directly corresponds to its low experimental binding to CD206+ macrophages (14 ± 2%) and its weak predicted affinity for the mannose receptor (pKdis = 4.0), supporting its alternative receptor targeting.

In contrast, the cyclic mannose derivative (ManCyc-PEI-FITC) exhibited substantial CD206 colocalization and distinct biochemical accumulation in FTIR maps, reflecting its strong cytometric binding efficiency (60 ± 7%). Ultimately, the branched triMan-GlcNAc2-PEI-FITC ligand displayed the most profound spatial colocalization with CD206 and the highest localized concentration of carbohydrate-protein complexes in FTIR mapping. This multimodal evidence seamlessly confirms its superior, highly specific targeting capacity, strictly matching its maximal predicted affinity for CD206 (pKdis = 7.4) and its peak experimental binding efficiency (80 ± 6%) observed via flow cytometry. Together, these multimodal findings provide robust validation of the ligands’ receptor-specific affinities.

### 2.3. Patient Cohort and Peripheral Blood and Bronchoalveolar Lavage Analysis

The overarching clinical cohort utilized in this study comprises 72 pediatric patients admitted with severe respiratory pathologies (detailed in Section 2.7). Initially, as a proof-of-concept that our approach based on the profiling of macrophage subpopulations can reliably evaluate the impact of pharmacological interventions on patient immune states, we selected a focal subset of 10 patients. These individuals shared a relatively homogeneous background of comparable obstructive respiratory conditions (asthma, bronchitis, and bronchiectasis) but presented with distinctly varying degrees of inflammation severity and contrasting prognostic risks.

This selection strategy ensured that drug-induced immune remodeling could be evaluated across a spectrum of distinct baseline immune states (Scores 0–5) while minimizing disease-specific confounding factors (Table 2). Consequently, the deliberate clinical heterogeneity within this 10-patient group was an intentional design choice; it aimed to elucidate how the drug’s mechanisms manifest across diverse initial immune landscapes, rather than to investigate the isolated pathogenesis of a singular disease.

Within this focused screening subset (age range 6–16 years), clinical diagnoses included bronchiectasis (*n* = 4), bronchial asthma (*n* = 5), and acute bronchitis (*n* = 1). Routine hematological assessments comprised a complete blood count (CBC) with a leukocyte differential (evaluating neutrophils, monocytes, and eosinophils) alongside serum C-reactive protein (CRP) quantification to establish baseline systemic inflammatory profiles (Table 2).

Using an unweighted binary scoring rubric (detailed in Section 4), these five blood parameters were integrated to calculate a cumulative clinical disease Severity Score (ranging from 0 to 5), which successfully stratified the cohort into Good (Score 0), Neutral (Scores 1–2), and Poor (Scores ≥ 3) prognostic groups. This stratification revealed significant heterogeneity in systemic inflammatory responses: while patients with a Score of 0 (P6, P8) exhibited physiological parameters and favorable prognoses, the majority of the cohort (*n* = 6) clustered into an intermediate Neutral group characterized by specific deviations, such as elevated CRP (P2, P4, P10; range 6.5–12.0 mg/L) or prominent leukocytosis (P3). High disease severity (Scores 3 and 5) was identified in P5—who presented with acute bronchitis, fever (38 °C), and marked CRP elevation (44.7 mg/L)—and P1, who exhibited bronchiectasis accompanied by severe leukopenia (WBC 2.16 × 10^9^/L) and monocytosis. Among these systemic profiles, eosinophil levels were specifically evaluated as a primary biomarker for Type 2 (T2) allergic airway inflammation [67,68,69,70]. Following standard clinical guidelines, fractions ≥ 5.0% typically indicate an allergic endotype, which often correlates with increased airway hyperresponsiveness and a higher likelihood of responding favorably to corticosteroid therapy. In our subset, true clinical eosinophilia was detected exclusively in P3 (6.2%), directly aligning with their bronchial asthma diagnosis, whereas distinct eosinopenia (<1.0%) was observed in P1 and P9, and other patients (P2, P5, P10) exhibited fractions merely at the upper limit of the normal range (4.4–4.6%).

So, here we have classified the selected cohort of patients according to degree of severity, according to systemic peripheral blood profiles, next we are going to correlate these parameters with the immune status of patients according to the profiles of macrophages subpopulations composition of proinflammatory, anti-inflammatory and intermediate phenotypes. We believe this will help to determine the risks of complications and dangerous conditions, as well as reveale the side effects of drugs on the immune status of patients

### 2.4. Example Profiles of Ligand Binding Patterns in BALF Macrophages: Clinical Prognoses and Doxycycline Modulation Effects

The application of carbohydrate-functionalized fluorescent ligands to clinical BALF samples provided a functional readout of alveolar macrophage states, extending significantly beyond the capabilities of conventional cytomorphology. These ligand-binding patterns reflect the functional status of specific carbohydrate-recognition domains, which differ markedly among M0, M1, and M2a phenotypes.

To transparently translate these cumulative, multidimensional fluorescence signals into quantitative biological metrics, we employed a linear least squares deconvolution approach with a non-negativity constraint. Mathematically, the binding intensities across the five distinct carbohydrate ligands (L1–L5), measured both in the presence and absence of the competitive inhibitor mannan, create a unique 10-dimensional “fingerprint” vector for each BALF sample. This method models the patient’s clinical vector as a linear combination of established standard basis vectors—specifically, the binding signatures of pure M0 (unpolarized), M1 (pro-inflammatory), and M2a (pro-resolving/allergic) reference macrophages validated in our previous work. By applying a Non-Negative Least Squares (NNLS) algorithm [38] we calculated the optimal coefficients required to reconstruct the patient’s BALF profile.

Crucially, this analysis yields the relative fractional contribution of each primary phenotype alongside a mathematical “residual” fraction. To evaluate the reconstruction quality of our linear model, the NNLS algorithm minimizes the unmapped Euclidean distance between the observed 10-dimensional clinical fingerprint and the reconstructed 3-component theoretical profile. The magnitude of this unexplained variance directly defines the residual fraction.

This residuals is not random noise, but rather a quantifiable metric of phenotypic plasticity. It absorbs marginal or atypical macrophage subpopulations (e.g., M2b, M2c, M2d) and, most importantly, intermediate transitional states that do not strictly align with the canonical tripartite basis. Because specific “atypical” polarization states (such as tumor-associated macrophages (TAM) or advanced immunosuppressive M2c cells) play a negligible role in the acute and subacute non-malignant pediatric respiratory diseases investigated here, aggregating them within the residual fraction is methodologically advantageous. It prevents mathematical overfitting and maintains the model’s analytical focus strictly on the dominant, disease-driving M1/M2a axis, while preserving the mathematical integrity of the underlying sample complexity. High baseline residuals therefore indicate a highly uncommitted, plastic immune microenvironment.

**Figure 4:**
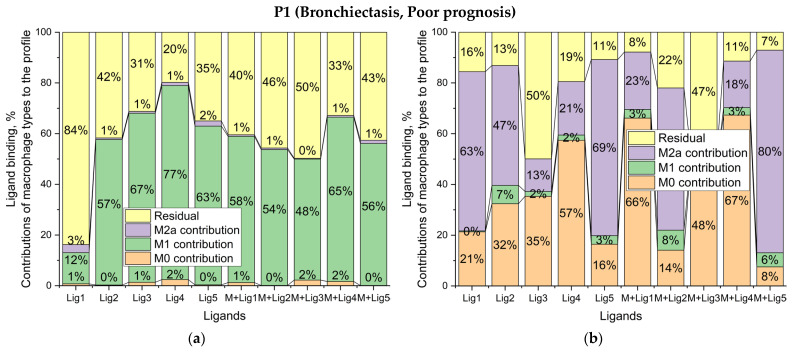

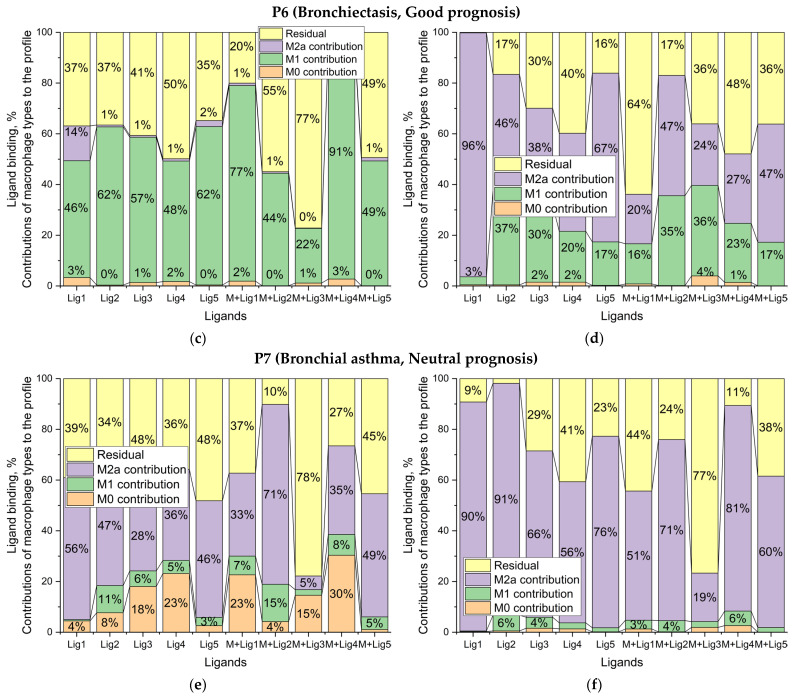
Deconvoluted ligand-binding profiles of BALF macrophages in pediatric respiratory disease patients before and after in vitro pharmacological remodeling. Vertical stacked bar charts display the relative percentage of each macrophage phenotype across the five carbohydrate-functionalized fluorescent probes (Lig1–Lig5). Data for competitive binding assays with mannan are labeled as M + Lig1 to M + Lig5. (**a**,**b**) Patient P1 (Diagnosis: Bronchiectasis, Poor prognosis) at baseline (**a**) and following treatment with doxycycline at 25 µg/mL (**b**). (**c**,**d**) Patient P6 (Diagnosis: Bronchiectasis, Good prognosis) at baseline (**c**) and following treatment with doxycycline at 25 µg/mL (**d**). (**e**,**f**) Patient P7 (Diagnosis: Bronchial asthma, Neutral prognosis) at baseline (**e**) and following treatment with doxycycline at 25 µg/mL (**f**). Color coding represents deconvoluted phenotype contributions: orange (M0, unpolarized/resident); green (M1, pro-inflammatory); purple (M2a, pro-resolving/remodeling); and yellow (Residuals, atypical/transitional subtypes). Percentage values within the bars indicate the calculated fractional contribution of each reference phenotype.

To test the clinical sensitivity of this method, we examined BALF macrophages from pediatric patients with distinct diagnoses and outcomes: P1 (bronchiectasis, poor prognosis), P6 (bronchiectasis, good prognosis), and P7 (bronchial asthma, neutral prognosis) (Figure 4). Patient P1 (bronchiectasis, poor prognosis, Figure 4a) presented with severe systemic inflammation, leukopenia, and elevated C-reactive protein. The BALF macrophage compartment was strongly M1-dominated (66%) with negligible M0 and M2a fractions (<1% each), while ligand binding to L1–L5 was largely suppressible by mannan, indicating high engagement of dysregulated carbohydrate-recognition receptors such as CD206. The relatively low residual component (32%) is consistent with a rigid, highly polarized inflammatory state.

In contrast, Patient P6 (bronchiectasis, good prognosis, Figure 4c) showed milder systemic inflammation and normal leukocyte counts, yet still exhibited an M1-biased profile (57%). Here, however, the residual fraction was markedly higher (41%), suggesting preserved phenotypic plasticity and the presence of intermediate or transitioning macrophage states, which aligns with the more favorable clinical trajectory. Patient P7 (bronchial asthma, neutral prognosis, Figure 4e) displayed a qualitatively different pattern, with a predominant M2a contribution (38%) and substantial residual (43%), in line with the alternative activation and tissue-remodeling programs characterizing Th2-driven asthma rather than acute bacterial infection.

To determine if this profiling framework could capture dynamic phenotypic shifts in response to pharmacological stimuli, we evaluated the effects of a clinically used antibiotic doxycycline (Figure 4b,d,f). Doxycycline was selected because it is a broad-spectrum tetracycline frequently prescribed for respiratory infections, and importantly, it possesses known immunomodulatory properties [49,50]. Within our developed test system and BALF macrophage model, doxycycline served as a good model compound to verify whether ligand-binding profil-based deconvolution can effectively resolve drug-induced macrophage repolarization.

To ensure that the observed phenotypic shifts reflected cellular repolarization rather than selective cytotoxicity, we rigorously assessed cell viability. At the dose of 25 µg/mL, doxycycline was entirely non-toxic, maintaining a high overall cell viability of >85% across all tested clinical samples. It is important to note that within the complex microenvironment of whole bronchoalveolar lavage fluid (BALF), the bioavailable concentration of the drug is likely attenuated due to substantial nonspecific binding to local protein and lipid constituents. Because the absolute viable cell pool remains stable during the assay, any significant alterations in the deconvoluted fractional profiles must indicate to active functional remodeling of the living cells’ surface receptor repertoire, rather than subset-specific cell death.

**Figure 5:**
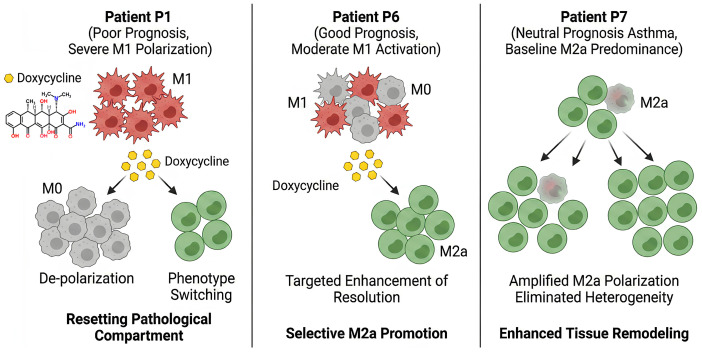
Doxycycline-induced macrophage phenotype responses in three pediatric respiratory disease patients. Schematics depict baseline BAL macrophage composition (top) and post-doxycycline (25 µg/mL) phenotype distribution (bottom) for each patient. Patient P1 (poor prognosis, bronchiectasis): baseline M1-dominant state transitions to M0-enriched and M2a-shifted phenotypes. Patient P6 (good prognosis, bronchiectasis): moderate baseline M1 activation selectively shifts toward M2a polarization with retained M0 components. Patient P7 (neutral prognosis, asthma): baseline M2a-predominant state intensifies to near-homogeneous M2a distribution with reduced heterogeneity. Doxycycline molecule (structural formula shown in the insert) indicates the exposure condition. Color coding: gray = M0 (resident), red = M1 (pro-inflammatory), green = M2a (resolution/remodeling).

Across the screening subset, doxycycline tends to suppress the M1 component (*p* < 0.01, paired Student’s *t*-test) and enhanced M2a contributions (*p* < 0.05). However, the functional implications varied based on the baseline state (Figure 4 and Figure 5). In the severe bronchiectasis case P1, the M1 fraction decreased from 66% to 4%, with concurrent increases in M0 and M2a fractions to 51% and 36%, respectively. In the milder case P6, doxycycline induced a targeted transition from M1 (57% to 27%) toward M2a (1% to 49%).

Conversely, in asthma patient P7, doxycycline exacerbated a preexisting M2a-skewed state, farther increasing the M2a fraction to 80% (*p* < 0.01). Because M2-like alveolar macrophages are known drivers of airway remodeling and fibrosis in chronic asthma, actively reinforcing this M2a polarization generates the clinically relevant hypothesis that such interventions might lead to risk aggravating structural lung damage rather than conferring a therapeutic advantage.

Notably, doxycycline treatment significantly reduced the residual M2 macrophage fraction across all patient samples, driving the residual population down to 9–23% (*p* < 0.05). This critical observation aligns seamlessly with established molecular mechanisms in the literature; specifically, it corroborates findings by Lokeshwar et al. [49], demonstrated that doxycycline effectively depletes proangiogenic macrophages (it is M2c-type), including Arginase-1 (Arg1)-positive subpopulations. So doxycycline may be used to enhance current antiangiogenic treatment approaches in neovascular age-related macular degeneration and in certain cancers. Consequently, our data support the paradigm that doxycycline suppresses the M2c-type of proangiogenic macrophages. Crucially, these findings underscore the fundamental importance of macrophage subpopulation profiles determination which is provided by test system proposed here rather than assessing the total M2 pool, as pharmacological agents exhibit highly specific immunomodulatory actions dependent on the precise cellular subpopulation.

Collectively, these case studies highlight the intrinsically dual nature of pharmacological immunomodulation (Figure 5). While doxycycline serves as a potent immunometabolic switch—dampening M1-driven inflammation in favor of M2a-associated tissue resolution—the ultimate clinical benefit of this shift is entirely contingent upon the baseline immune microenvironment. For instance, while this repolarization offers robust therapeutic potential in M1-dominated bronchiectasis (P1, P6), it risks becoming highly maladaptive in M2a-biased asthma (P7), where further skewing could exacerbate the allergic endotype.

Moreover, in light of the aforementioned anti-angiogenic effects mediated by M2c depletion, the stakes of untargeted administration are even higher. Altering the M2c pool can profoundly impact local vascular networks, meaning that “blind” therapy could inadvertently disrupt essential tissue repair or trigger pathological remodeling depending on the disease stage.

Consequently, our ligand-based BALF macrophage phenotyping emerges not merely as an exploratory method, but as a critical diagnostic prerequisite. By distinguishing patients who stand to benefit from targeted M1 or M2c modulation from those vulnerable to treatment-induced complications—such as asthma exacerbations, aberrant angiogenesis, or progressive fibrosis—this framework underscores the absolute necessity of profiling the localized macrophage system prior to therapy.

### 2.5. Ex Vivo Screening of Drug-Compounds for Remodeling Macrophage Functional States in Patient BALF

**Figure 6:**
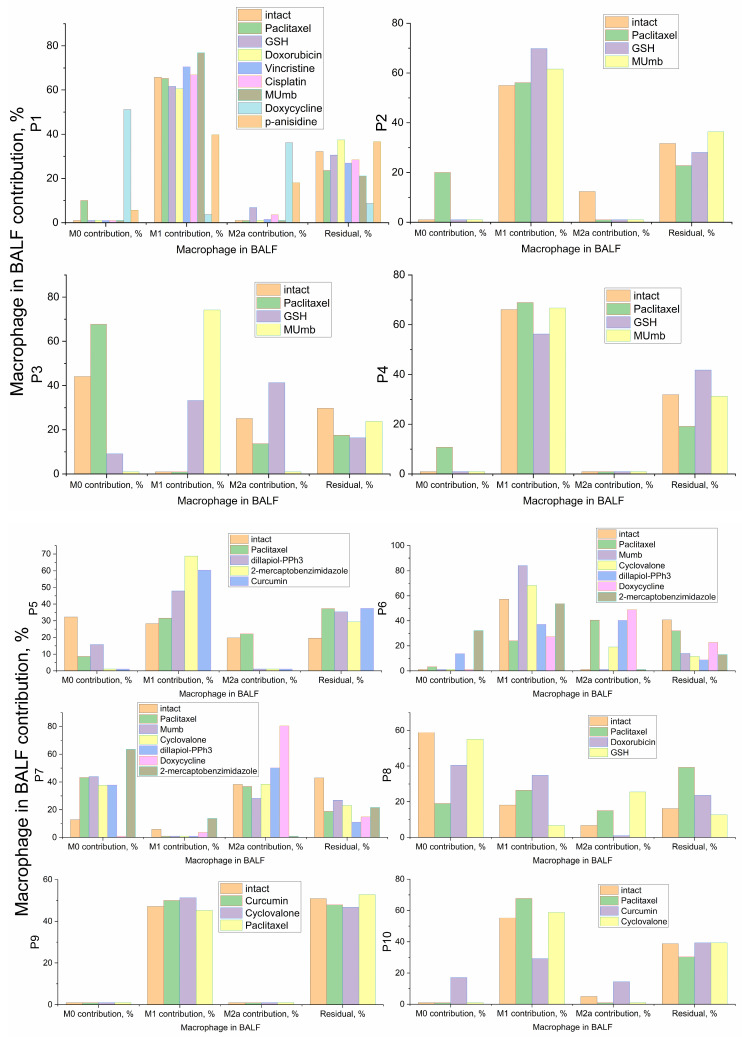
Individual remodeling of macrophage functional states in patient BALF. Bar charts illustrate the percentage contribution of M0 (unactivated), M1 (pro-inflammatory), M2a (anti-inflammatory/repair), and Residual classes for each patient under “intact” conditions and following incubation with various bioactive substances.

Building upon the distinct baseline macrophage profiles identified in Section 2.4, we conducted an ex vivo screening of a targeted drug-compound library to evaluate how standard antibiotics and cytostatics effects on macrophages depending on the initial immune status, type and severity of the disease. The tested pharmacological compounds were classified by their primary mechanisms of action and structural categories, as detailed in Table 3 and Table S2. The overarching modulatory effects of these categorized agents across the patient cohort are summarized in Figure 6.

Analysis of intact (untreated) alveolar macrophages revealed profound inter-patient heterogeneity, providing a clear molecular rationale for why standardized therapeutic protocols often yield inconsistent clinical outcomes (Table S1, Figure 6). For example, patients with chronic inflammatory conditions, such as bronchiectasis (P1 and P4), exhibited a baseline phenotype heavily skewed toward the pro-inflammatory M1 state. In these cases, the M1 fraction (up to 66%) was significantly higher than the M0 or M2a fractions (*p* < 0.01), indicating a “locked-in” inflammatory profile. Conversely, patients P3 and P8 presented with a quiescent profile dominated by unactivated M0 cells (44% and 59%, respectively) with negligible M1 activity (<18%). This marked baseline divergence implies that while patients like P1 require immune dampening, patients like P3 would likely benefit from targeted immune stimulation—a critical nuance lost in generic treatment guidelines.

To test the feasibility of controlled immune reactivation in quiescent phenotypes, we evaluated 4-Methylumbelliferone (MUmb). MUmb is a derivative of the coumarin family, a widely distributed natural scaffold found in plants of the Apiaceae family (e.g., carrot, coriander, and garden angelica). Given the known immunostimulatory properties associated with coumarin derivatives, we hypothesized that MUmb would effectively polarize dormant M0 cells into an active M1 state. As demonstrated in Figure 6, the data provided robust statistical validation of this hypothesis. In Patient P3, whose intact macrophages were predominantly unactivated, incubation with MUmb triggered a substantial phenotypic inversion, driving the M1 contribution to 74% (*p* < 0.001 compared to the untreated control; Table S1). A similar reinforcement of the M1 phenotype was observed in Patient P6 (M1 increased from 57% to 84%, *p* < 0.01) and Patient P1 (from 66% to 77%, *p* < 0.05). These ex vivo findings align with previous in vivo animal studies, which demonstrated that umbelliferone administration effectively modulates allergic and immune airway alterations induced by asthmatic processes [71].

Conversely, for patients exhibiting hyper-inflammatory baselines, the therapeutic imperative is to suppress M1 dominance and promote resolution. In this context, our screening highlighted the marked efficacy of the antioxidant glutathione and the phenylpropanoid dillapiole. As illustrated in the resulting profiles (Figure 6), treatment with these agents effectively mitigated the acute inflammatory load, driving a pronounced reduction in the M1 fraction while concomitantly increasing the resolving M0 and M2a populations. Both glutathione and dillapiole provided a robust counter-regulatory effect, establishing them as highly promising candidates for dampening respiratory inflammation. This potent anti-inflammatory shift parallels the repolarization observed with doxycycline, which similarly abolished M1 expression in favor of M2a phenotypes in hyper-inflammatory samples (as discussed extensively in Section 2.4). In sharp contrast, agents such as paclitaxel failed to induce significant remodeling, generally maintaining the pre-existing inflammatory profile (e.g., Patient P4 maintained a 69% M1 fraction, *p* > 0.05 vs. intact control).

### 2.6. Statistical Validation and Diagnostic Applicability of Pharmacological Remodeling

**Figure 7:**
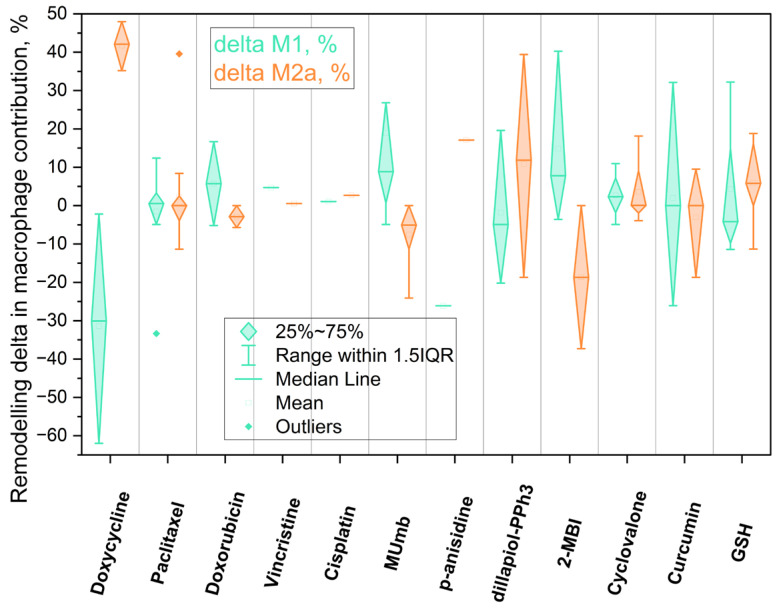
Statistical analysis of macrophage remodeling capacity. Violin and box plots representing the distribution of changes (delta, %) in M1 (green) and M2a (orange) contributions relative to the intact state across the patient cohort. The box indicates the interquartile range (IQR), the horizontal line represents the median, and the small square represents the mean.

To quantify the immunomodulatory potential of the tested library across the patient cohort, we analyzed the remodeling delta (Δ, %), defined as the net shift in phenotype contribution between treated and intact states (Figure 7).

Using least-squares deconvolution and pattern recognition of patient-derived BALF cell profiles against established M0, M1, and M2a reference populations, our ex vivo platform translates complex repolarization events into a measurable net clinical vector (Table 3). To systematically evaluate the secondary immunomodulatory effects of these agents—while carefully accounting for inherent differences in their primary mechanisms of action and applied dosages—we stratified the screened compounds into three distinct functional and pharmacological categories: antibiotics, cytostatics, and immunomodulatory adjuvants.

#### 2.6.1. Antibiotics Secondary Effects

Among the evaluated compounds, doxycycline emerged as the most potent directional driver of macrophage repolarization in our study. It induced a profound reduction in the pro-inflammatory M1 fraction (the entire interquartile range for ΔM1 remained below zero, *p* < 0.001) alongside a concomitant expansion of the M2a population (*p* < 0.01). This marked immunometabolic shift is best exemplified by a ~60% decrease in M1 expression in Patient P1 and an 80% surge in the M2a fraction in Patient P7.

While standard isolated cell-line models occasionally report that doxycycline inhibits general IL-4/IL-13-induced M2 polarization, this apparent contradiction is resolved by examining specific macrophage subcategories. As discussed in Section 2.4, doxycycline selectively suppresses proangiogenic macrophages, including Arginase-1-positive subsets (mainly M2c) [49]. Consistent with this mechanism, the depletion of the M2c pool in our BALF by doxycycline resulted to remodulate the residual alveolar macrophages toward the reparative M2a state. This dynamic clearly illustrates the critical gap between broad in vitro models and the complex cytokine milieu of patient-derived material. It reinforces a fundamental methodological conclusion: the profiling of distinct sub-phenotypes (e.g., M2a vs. M2c) is absolutely essential, as treating the “M2 pool” as a single entity masks critical pharmacological effects.

The pronounced M2a-promoting capability of doxycycline demonstrates how a single antibiotic can exert either beneficial or detrimental secondary effects dictated strictly by the patient’s baseline immune context. In Th2-dominated allergic phenotypes, such as bronchial asthma, the localized environment is already biased toward a maladaptive M2 response; administering an M2a-promoting agent in this context inadvertently risks exacerbating airway remodeling and fibrosis. In stark contrast, for patients with chronic M1-driven inflammation (e.g., bronchiectasis), this exact repolarization profile becomes a highly desirable therapeutic mechanism to drive tissue resolution. Detecting and anticipating this duality at the individual patient level is precisely the diagnostic purpose of our ex vivo test system.

#### 2.6.2. Cytostatics Secondary Effects

The four tested cytostatics produced markedly different secondary immunomodulatory signatures. Paclitaxel stood apart by shifting macrophages toward the M1 or M0 state, indicating a functional passivation of the cells. While low-dose paclitaxel is known to promote M1 polarization via TLR4/NF-κB signaling, our system demonstrated a dominant suppression of polarization markers. This effect is likely driven by STAT3 inhibition. The high inter-patient variance observed for ΔM2a (*p* > 0.05) indicates to context-dependent properties of paclitaxel as a modulator depending on the patient’s baseline profile.

Conversely, doxorubicin treatment predominantly shifted the functional profile toward an M1 state, accompanied by a reduction in the M2a fraction. However, this effect is profoundly dose- and context-dependent. As highlighted in our previous study [38], under certain conditions, doxorubicin promote generic M2 polarization. Specifically, it drives the M2c and M2d phenotypes—which in our current analytical framework manifest precisely as an expanded “residual M2” pool. This repolarization pathway is clinically critical, as it can activate TAMs and inadvertently provoke secondary metastasis. We corroborated this phenomenon experimentally, demonstrating that specific doxorubicin formulations can trigger a distinct M2-promoting effect. These findings vividly illustrate the critical importance of drug formulation: the delivery vehicle itself can dictate the directional trajectory of secondary immunomodulation. Consequently, while modifying nanocarriers or formulations offers a powerful strategy to intentionally guide immune responses, these off-target effects remain highly specific to both the individual patient and the applied formulation. To ensure therapeutic safety and prevent maladaptive TAM activation, personalized ex vivo screening of these formulations is an absolute clinical prerequisite.

Finally, vincristine and cisplatin exerted a minimal effect on the overarching macrophage phenotype. For both agents, the violin plot distributions remained tightly clustered near zero for both ΔM1 and ΔM2a, indicating that, at the evaluated concentrations, these compounds do not substantially remodel the baseline macrophage landscape for the cohort of pediatric patients with obstructive respiratory pathologies (asthma, bronchitis, and bronchiectasis). This distinct absence of a secondary immunomodulatory signal is diagnostically relevant in its own right; it clearly distinguishes vincristine and cisplatin from agents like doxorubicin and paclitaxel, positioning them as cytostatics with a significantly lower risk of general off-target immune perturbation.

#### 2.6.3. Adjuvants and Small Molecules as Context-Dependent Modulators

**4-Methylumbelliferone (MUmb)**, a coumarin-based hyaluronan synthesis inhibitor, was the most consistent immunostimulant. ΔM1 showed a tightly clustered positive shift (*p* < 0.05), in line with its reported ability to polarize macrophages toward an M1 profile. In our test system, MUmb represents a compound capable of activating quiescent macrophages—a key property for reversing immuno-silence what is referred to as a problem in pulmonary tuberculosis and potentiate antitumor (anty TAM) immunity-activator.

**Curcumin** exhibited a pronounced antioxidant and immunoregulatory properties: it attenuated the M1 phenotype in patients with a high inflammatory baseline (e.g., P10) while paradoxically promoting M1 polarization in those with lower baseline inflammation (e.g., P5). This aligns with recent findings highlighting the complex, pleiotropic effects of turmeric-derived compounds on macrophages; for instance, Curcuminoids have been shown to simultaneously downregulate M1 surface markers (CD86) and upregulate M2-associated markers (CD163, IL-10, TGF-β), while actively enhancing the expression of antibacterial pro-inflammatory cytokines (IL-1β, IL-6, TNF-α) [72]. **Cyclovalone**—a synthetic curcuminoid characterized by cyclooxygenase-inhibitory activity—elicited only weak and variable modulation, driving moderate M2a induction in a subset of patients while merely maintaining the M1 phenotype in others.

**2-Mercaptobenzimidazole (2-MBI)**, a benzimidazole derivative with documented antimicrobial and analgesic properties, demonstrated strong M1 induction in certain patient profiles (e.g., P5). In contrast, p-Anisidine and Dillapiol-PPh3—a mitochondria-targeted terpenoid–triphenylphosphonium conjugate previously characterized by our group [73]—consistently promoted an M2a phenotypic shift, firmly positioning them as candidate anti-inflammatory adjuvants.

**Glutathione (GSH)** exhibited a consistent ability to attenuate the pro-inflammatory M1 fraction across the patient cohort (Figure 7). This finding aligns seamlessly with its established biological role as a critical cellular redox buffer [34]. Because M1 macrophage activation and maintenance are heavily dependent on reactive oxygen species (ROS) signaling and oxidative stress, the exogenous application of GSH acts as a potent antioxidant, neutralizing the oxidative load required to sustain hyperinflammation. Consequently, GSH functions as a highly effective homeostatic stabilizer, safely dampening the M1-driven inflammatory response.

The functional divergence observed among the evaluated adjuvants underscores the critical necessity for disease-specific immunomodulatory strategies. Distinct clinical states present fundamentally opposing requirements: conditions such as severe pneumonia or acute asthma necessitate the rapid suppression of hyperinflammation, whereas post-infectious immunoparalysis demands targeted macrophage reactivation. Our ex vivo findings directly address these therapeutic targets. MUmb consistently drove robust M1 polarization, highlighting its potential for reversing immunoparalysis. Conversely, compounds such as **p-anisidine** and **dillapiole-PPh_3_**—alongside the redox-buffering action of GSH—exhibited pronounced anti-inflammatory properties, significantly suppressing M1 fractions and promoting reparative M2a polarization. Curcumin, however, induced highly context-dependent responses, mandating personalized screening prior to administration.

Finally, presented stratification reveals that pharmaceutical agents within the same therapeutic class can exert diametrically opposed secondary immunomodulatory effects. By profiling these localized cellular responses, our ex vivo diagnostic platform provides a rational, data-driven framework to tailor adjuvant selection to the patient’s specific baseline immune state, effectively transitioning respiratory therapy from empirical antimicrobial regimens toward precision immune synchronization.

#### 2.6.4. The Translational Bridge to Precision Immunology

Recent comprehensive studies [25] have employed highly complex, multi-method approaches to map how specific macrophage subpopulations (such as pro-fibrotic CCRL2+ or reparative CD160+ cells) shift during acute inflammation in organs like the liver or heart. However, the sheer volume of high-dimensional data generated by these techniques often creates a convoluted and overwhelming picture. Rather than yielding clear, actionable insights, this hyper-granularity makes it exceedingly difficult to extract straightforward, practical conclusions that can be directly applied to bedside decision-making.

Our ex vivo test platform provides a vital, rapid alternative: tracking the immunomodulatory vector via functional pattern deconvolution. Because a primary therapy (like an antibiotic or cytostatic) can have contradictory immune side-effects depending on the patient’s baseline context, empirical treatment is no longer sufficient. By utilizing this diagnostic platform, clinicians can proactively identify whether a patient is heading toward hyper-inflammation or immunoparalysis. Consequently, primary therapies can be precisely “complemented” with targeted adjuvants or specific formulations to correct the immune trajectory, shifting the paradigm from simple pathogen eradication to personalized immune synchronization.

### 2.7. Multivariate Linear Discriminant Analysis Reveals Distinct Ligand-Binding Signatures Correlated with Disease Severity

Deconvolution analysis assessing the contributions of the M0/M1/M2 to the macrophages profile for 10 selected patients (Table 2) revealed that drugs of various categories can significantly remodel the macrophage polarization in a complex, context-dependent way. A single agent may resolve inflammation in one host while exacerbating pathology in another depending on the baseline immune status. We reasoned that multiple ligand binding data studied here reflects a much more complex landscape of macrophage status. Being processed by multivariate mathematical approach, this data can yield clinically significant prognosis for every individual patient.

**Figure 8:**
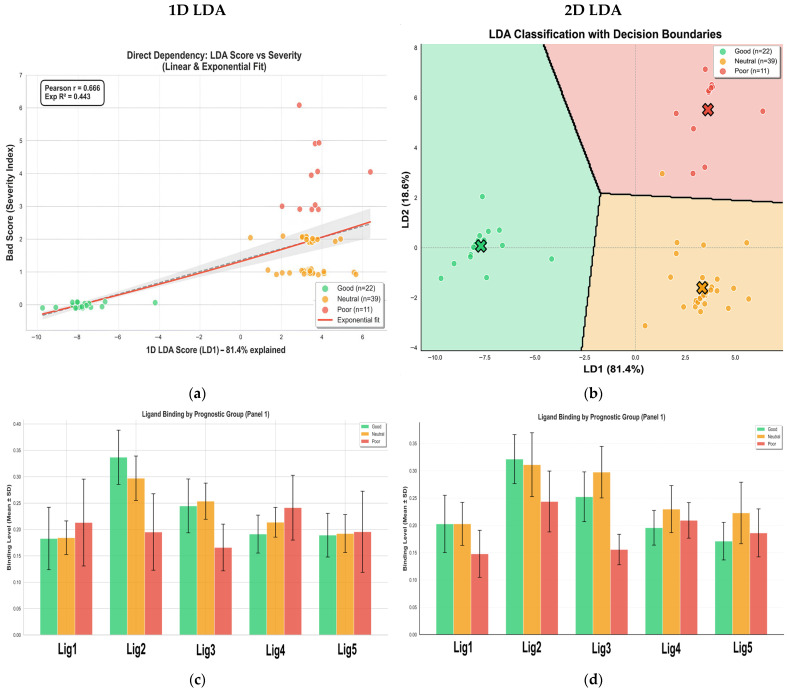

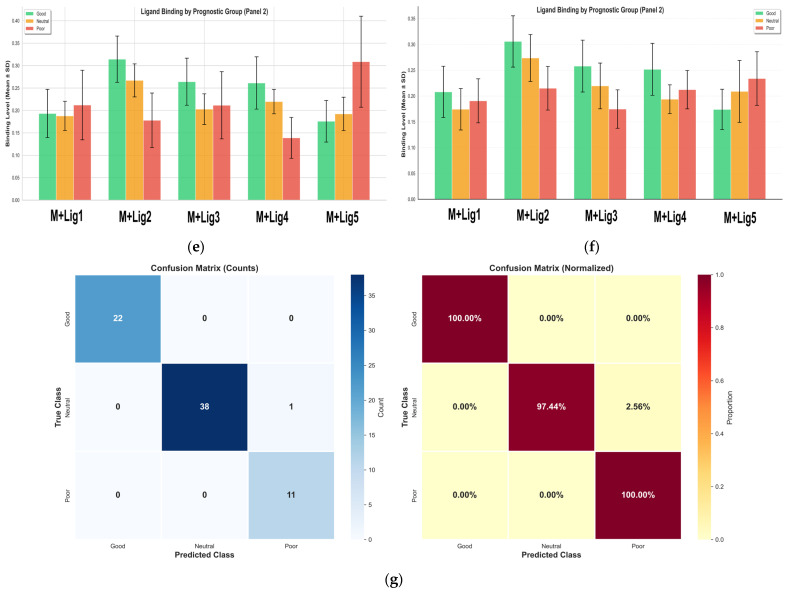
Multivariate Linear Discriminant Analysis (LDA) of comprehensive BALF ligand-binding profiles and their correlation with clinical disease severity. (**a**) Correlation analysis between the unidimensional LDA Score (LD1) and the composite clinical Severity Index. The regression line (red) with 95% confidence intervals (gray) reveals a strong dependency (Pearson *r* = 0.666). (**b**) 2-dimensional projection (LD1 vs. LD2) of the LDA demonstrating maximal structural resolution and clustering of the specific prognostic cohorts (Good, Neutral, and Poor). The crosses serve as markers for the centres of the respective zones. The dotted gray lines represent zero values on both the ordinate and the abscissa. (**c**,**e**) Raw ligand binding levels (Mean ± SD) for individual ligands (Panel 1) and Mannan-inhibited conditions (Panel 2) utilized as inputs for the 1D LDA model. (**d**,**f**) Corresponding raw binding data utilized for the 2D LDA model. (**g**) Confusion matrices displaying the robust classification performance and accuracy of both LDA models across the defined clinical cohorts.

We applied Linear Discriminant Analysis (LDA) to the aggregated ligand-binding data. This model was trained to segregate the 72-patient population into “Good,” “Neutral,” and “Poor” prognostic cohorts using a composite Severity Index derived from standard leukocyte parameters and C-reactive protein levels (Figure 8).

To comprehensively evaluate the diagnostic potential of our platform, we analyzed both 1-dimensional (1D) and 2-dimensional (2D) LDA projections. As depicted in the 2D projection (Figure 8b) and the corresponding confusion matrices (Figure 8g), utilizing both the first (LD1) and second (LD2) linear discriminants yields the highest classification accuracy. The LD2 axis captures secondary variations within the dataset, allowing for tighter spatial clustering of the prognostic groups. This 2D model effectively demonstrates the maximal mathematical and biological resolving power of our comprehensive ligand panel.

However, for the subsequent downstream analysis and practical clinical translation, we deliberately focused on the 1D LDA score (LD1, Figure S5). In discriminant analysis, the first discriminant function (LD1) is mathematically designed to capture the overwhelming majority of the variance that separates the defined classes. As shown in Figure 8a, the LD1 score alone acts as the primary axis of disease trajectory, serving as a robust, continuous unidimensional biomarker that strongly correlates (Pearson r = 0.666) with the clinical Severity Index. By collapsing the complex, multidimensional ligand-binding data into a single numerical index (the LD1 score), we provide a highly translatable “thermometer” of macrophage dysregulation. This single metric can be easily and intuitively monitored during pharmacological interventions in a clinical setting, avoiding the complexity of interpreting dual-coordinate shifts while retaining the core predictive power of the assay.

The resulting classification performance was highly robust, as evidenced by the confusion matrices (Figure 8g). The model achieved high sensitivity for the extreme phenotypes, correctly identifying all patients in the “Good” (*n* = 22) and “Poor” (*n* = 11) categories, with only a single misclassification occurring in the “Neutral” group (97.44% accuracy). This statistical separation is visually represented in the two-dimensional canonical space (Figure 8b), where the first linear discriminant (LD1) captures 60.5% of the variance. The topological distribution within this space is clinically revealing: the “Good” prognosis group (green) forms a tight, cohesive cluster. This does not necessarily represent a naive homeostatic baseline, but rather a distinct, regulated immune state indicative of a correct therapeutic trajectory and a high probability of recovery. In sharp contrast, the “Poor” prognosis group (red) exhibits significant spatial dispersal. This loss of clustering density reflects high phenotypic heterogeneity, suggesting that severe pathology is characterized not by a single resistant phenotype, but by a chaotic loss of immune regulation and a drift into stochastic dysregulation.

Crucially, the LD1 axis functions as more than a categorical classifier; it serves as a continuous biological meter of macrophage dysfunction. As shown in the regression analysis (Figure 8a), there is a significant positive correlation (Pearson *r* = 0.666) between the LD1 score and the clinical Severity Index. The relationship is best described by an exponential fit (R^2^ = 0.443), implying that as the macrophage phenotype deviates from the favorable therapeutic zone (increasing LD1), clinical deterioration does not merely increase linearly but accelerates. This suggests that the LD1 score captures the underlying biological substrate of disease progression before it manifests as catastrophic clinical failure.

To decode the molecular drivers of this classification, we examined the raw and inhibited ligand binding profiles (Figure 8c–f and Figure S6). The bar charts illustrate distinct binding signatures across the three groups for both native conditions (Panel 1) and Mannan-inhibited conditions (Panel 2, labeled M + Lig). Notably, the feature importance hierarchy indicated a dominance of inhibition by mannan terms. This points to the functional integrity of the Mannose Receptor (CD206) as a primary discriminator. Unlike standard antibody staining, which provides a binary “digital” signal (presence/absence), our library of medium-affinity ligands generates a nuanced “analog” readout of receptor functionality.

In patients with favorable prognoses, macrophage binding profiles demonstrate specific, high-affinity interactions responsive to competitive inhibitors (Figure 8c,e). In “Poor” prognosis cohorts, this target specificity is lost. The aberrant binding in severe cases (Figure 8d,f), particularly under inhibited conditions, indicates non-specific uptake or receptor saturation. Therefore, the binding profile acts as an early-warning biomarker: elevated LD1 scores warrant aggressive monitoring or treatment adjustments, even if clinical symptoms remain mild.

This discriminant model also provides a quantifiable therapeutic target. Because macrophage polarization drives the local cytokine milieu, evaluating treatment success by downstream cytokine reduction alone is insufficient. Instead, an effective intervention must reposition the patient’s cellular state on the LDA plot, shifting the profile from the dysregulated “red” zone to the “green” resolution cluster.

**Figure 9:**
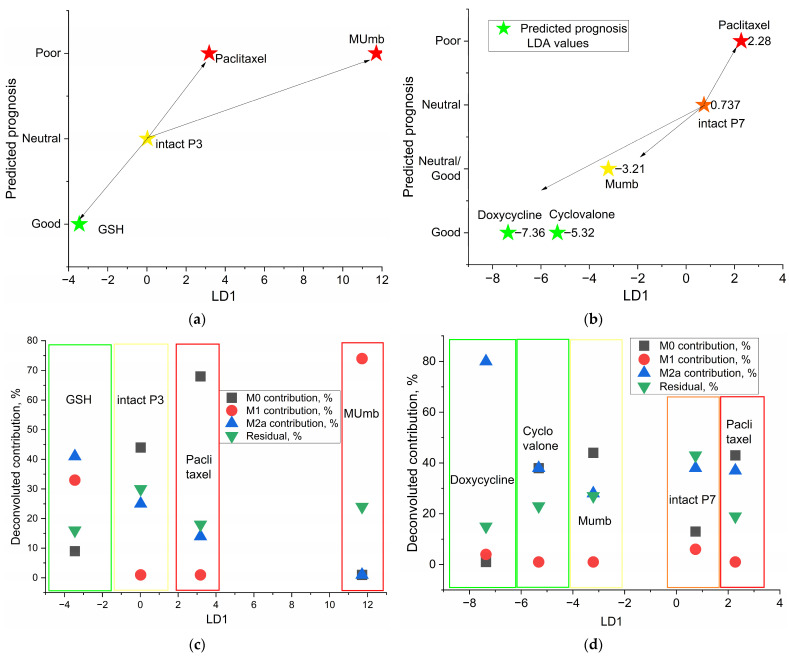
Vector analysis of pharmacological remodeling in patient-specific BALF cells populations. (**a**,**b**) Projection of ex vivo treated alveolar macrophages onto the canonical LDA space. Arrows indicate the magnitude and direction of the therapeutic vector from the intact state (start) to the remodeled state (end). Stars represent the predicted prognosis zone: Green (Good), Yellow (Neutral), Red (Poor). (**a**) Trajectories for Patient P3 (initially Neutral). (**b**) Trajectories for Patient P7 (initially Neutral/Poor). (**c**,**d**) Corresponding deconvoluted phenotype contributions (M0, M1, M2a) for the specific LD1 coordinates shown above for Patient P3 (**c**) and Patient P7 (**d**). Colored boxes indicate predicted prognosis zone: Green (Good), Yellow (Neutral/good), Orange (Neutral), Red (Poor).

Importantly, the variation in LD1 status shown in Figure 9 reflects the direct ex vivo application of the drugs to a single BALF sample taken at a specific timepoint. The observed shift in LD1 coordinates demonstrates the drug’s immediate capacity to remodel the macrophage phenotype within the in vitro assay, rather than tracking a longitudinal in vivo clinical recovery of the patient. This distinction confirms that the agents directly alter the sample’s LD1 status, validating the platform as a predictive diagnostic screening tool rather than a monitor of disease resolution.

Having established the static topological landscape of disease severity, we advanced to the critical phase of the analysis: visualizing the dynamic shifts induced by therapeutic agents. By projecting the ex vivo remodeling data onto the canonical LDA space defined in Figure 8, we calculated the specific “therapeutic vector” generated by each compound. This approach effectively overlays pharmacological trajectories onto the patient’s specific immune landscape, allowing us to determine whether a drug drives the macrophage population toward the homeostatic “Good” cluster or propels it further into the dysregulated “Poor” zone. This vector analysis is detailed in Figure 9, which contrasts the distinct pharmacological responses of two patients with divergent baseline statuses—Patient P3 (Neutral) and Patient P7 (Borderline/Poor).

The primary objective of this ex vivo modeling is not to simulate the targeted treatment of specific clinical conditions, but rather to utilize a highly tractable platform to ascertain how various pharmacological agents fundamentally alter macrophage status and dictate resulting clinical trajectories.

To bridge the gap between macroscopic predictive outcomes and their underlying cellular mechanisms, Figure 9c,d map the deconvoluted phenotypic fractions (M0, M1, and M2a) directly against the primary discriminant axis (LD1). Derived from the least-squares deconvolution of localized ligand-binding profiles, this complementary visualization is analytically signiifcant. While the LDA diagrams (Figure 9a,b) establish the directional vector of prognosis, the compositional profiles (Figure 9c,d) reveal the exact cellular restructuring driving those macroscopic shifts. This allows for the crucial differentiation of mechanisms: it elucidates whether a drug-induced shift toward a “Poor” prognosis is driven by an aggressive hyper-inflammatory spike or by profound functional immune exhaustion.

The trajectory analysis for Patient P3 (Figure 9a,c) acutely illustrates the absolute necessity of such personalized screening. Initially positioned within the “Neutral” baseline zone (LD1 ≈ 0), this patient’s sample exhibited a robust restorative shift under the influence of adjuvant glutathione (GSH), which drove the clinical vector deep into the “Good” prognostic territory (LD1 < −3). Phenotypically, this resolution directly correlated with a resurgence of the reparative M2a fraction and a concomitant suppression of the unactivated M0 population (Figure 9c). Exposing the same baseline profile to different compound classes, however, revealed distinctly divergent mechanisms of cellular destabilization. For instance, the immunomodulator MUmb provoked a massive, maladaptive spike in the pro-inflammatory M1 fraction (reaching ~75%), it drove this patient’s vector sharply toward the “Poor” prognosis zone with cytokine storm-like state. While such a pro-inflammatory M1 response would be therapeutically advantageous in contexts requiring robust immune clearance—such as tuberculosis or the targeting of tumor-associated macrophages (TAMs)—it is strictly detrimental in this specific respiratory context. Evaluated independently, the cytostatic agent paclitaxel also proved highly deleterious; it directed the vector toward the “Poor” zone not via inflammation, but by inducing profound functional deactivation, characterized by a dominant, marker-negative M0 phenotype.

A fundamentally different therapeutic imperative emerged for Patient P7 (Figure 9b,d), whose baseline status was already severely compromised. While administered primarily as an antibacterial agent, doxycycline concurrently functioned as a highly potent “resolution driver” for the localized immune system. It generated a corrective vector of significant amplitude (LD1 = −7.36) that successfully repositioned the cellular phenotype within the “Good” prognostic cluster. As confirmed by the complementary deconvolution data (Figure 9d), this systemic restoration was underpinned by a decisive polarization toward the M2a phenotype (~80%). Among the evaluated adjuvants, cyclovalone similarly demonstrated a favorable, albeit less aggressive, corrective trajectory. The independent assessment of paclitaxel once again confirmed its consistent destabilizing potential (LD1 = 2.28), exacerbating the patient’s existing immune dysregulation. We validated the LD1 axis as an integral, mechanistically grounded biomarker: negative coordinate shifts signify robust immune consolidation and tissue resolution, whereas positive trajectories reflect an entropic drift toward either maladaptive hyper-aggression or dangerous functional inertia.

## 3. Discussion

The conventional paradigm of respiratory disease management predominantly focuses on pathogen eradication or generalized immunosuppression, frequently overlooking the profound secondary immunomodulatory effects that pharmacological agents exert on the host’s localized innate immune landscape. The present study addresses this critical gap by introducing a personalized ex vivo profiling platform designed to decode and therapeutically remodel the functional states of bronchoalveolar lavage fluid (BALF) macrophages.

Historically, macrophage phenotyping has relied heavily on systemic cytokine panels or binary surface marker analysis (e.g., conventional CD206 or CD86 antibody staining). Beyond being methodologically cumbersome and prohibitively expensive for routine screening, these approaches lack direct translational applicability at the clinical bedside. More importantly, isolated cytokine quantification fails to capture the complex, shifting balance of the localized immune network; evaluating the complete compositional profile of macrophage subpopulations is fundamentally more informative than measuring downstream signaling molecules. To overcome these limitations, our platform based on the carbohydrate-functionalized fluorescent ligands coupled with an AI-driven analytical solution. This method enables the precise identification of the secondary immunomodulatory effects of conventional drugs. As demonstrated, rather than merely confirming the presence or absence of a receptor (as it is realized in the case of antybodyes- based analisys), these ligands provide an “analog” readout of functional binding capacity and spatial receptor clustering. This multidimensional binding signature strictly correlates with both the overarching macrophage subpopulation profile and the patient’s individual clinical trajectory.

A primary conceptual advancement of this study is the empirical demonstration of context-dependent pharmacological remodeling. The functional screening establishes that the immunomodulatory vector of a given drug is not solely an intrinsic property of the molecule, but rather a dynamic outcome dictated entirely by the patient’s baseline immune microenvironment. For instance, while a broad-spectrum antibiotic such as doxycycline acts as a potent resolution driver in an M1-dominated profile, it risks exacerbating immune dysregulation within an M2a-skewed environment. This profound context-dependency extends across distinct pharmacological classes. We demonstrated that antioxidant therapies (such as glutathione) and specific adjuvants function as critical homeostatic stabilizers, actively dampening acute oxidative stress and suppressing hyper-inflammatory M1 dominance. Conversely, immunostimulatory agents like the coumarin derivative umbelliferone (MUmb) aggressively drive M1 polarization—a mechanism that is highly beneficial for reversing post-infectious immunoparalysis, yet potentially deleterious in acute inflammatory states.

To translate these complex, multidimensional phenotypic shifts into actionable clinical insights, we employed Linear Discriminant Analysis (LDA) integrated with a deliberately constrained mathematical basis representation (M0, M1, and M2a) within our deconvolution algorithm. By focusing strictly on these primary pathophysiological axes—acute inflammation (M1) and allergic/tissue-remodeling responses (M2a)—the model effectively circumvents mathematical overfitting. It compels the extraction of definitive, clinically actionable polarization vectors, intentionally relegating atypical or uncommitted cellular subsets to a calculated residual fraction. Consequently, the resulting one-dimensional LDA score (LD1) functions as a robust, continuous biological “thermometer.” It detects latent macrophage dysregulation and tracks the dynamic M1/M2a balance, discriminating primary therapeutic targets from peripheral phenotypic noise.

While the proposed profiling platform establishes a robust diagnostic framework for personalized medicine, the disease-specific pharmacological remodeling trajectories presented herein should be viewed as preliminary demonstrations of the platform’s analytical capability. Future validation across larger, stringently stratified patient cohorts is requisite to translate these findings into standardized, precision-guided therapeutic protocols.

## 4. Materials and Methods

### 4.1. Reagents

Polyethyleneimine (PEI, MW 1.8 kDa) was purchased from Sigma-Aldrich (St. Louis, MO, USA). Carbohydrates, including mannan, α-D-mannose (Man), methyl-α-D-mannoside (Me-Man), D-galactose, and D-lactose, were obtained from Sigma-Aldrich (St. Louis, MO, USA). Mannotriose-di-(N-acetylglucosamine) (triMan-GlcNAc) was obtained from Dayang Chem Co. (Hangzhou, China). Fluorescein isothiocyanate (FITC) was purchased from Sigma-Aldrich (St. Louis, MO, USA). Therapeutic agents, including paclitaxel, doxorubicin, vincristine, cisplatin, glutathione (GSH), 4-methylumbelliferone (MUmb), curcumin, doxycycline, p-anisidine, 2-mercaptobenzimidazole, listed in Table 3, were obtained from commercial sources and dissolved in appropriate solvents. All other chemicals and solvents were of analytical grade and used without further purification.

### 4.2. Synthesis of Carbohydrate-Functionalized Fluorescent Ligands

A panel of five fluorescent ligands (L1–L5) was synthesized by conjugating specific carbohydrate moieties to a polyethyleneimine (PEI, 1.8 kDa) backbone labeled with fluorescein isothiocyanate (FITC), in accordance with our previously established protocols [37,74,75]. The synthesis proceeded via a sequential two-step strategy designed to optimize both fluorescence intensity and receptor recognition specificity.

First, the fluorescent precursor (PEI-FITC) was prepared by reacting PEI with FITC in anhydrous dimethyl sulfoxide (DMSO). The reaction was conducted in the dark at room temperature for 12 h to achieve a PEI:FITC molar ratio of approximately 1:1, ensuring distinct labeling without compromising polymer solubility.

Subsequently, carbohydrate functionalization was performed using two distinct coupling chemistries selected based on the structural requirements of the target ligand.

**Reductive Amination (Ligands L1, L3, L5).** For linear mannose (ManLin, L1), linear galactose (GalLin, L3), and the branched trimannoside derivative (triMan-GlcNAc2, L5), conjugation was achieved via reductive amination. The specific carbohydrate was dissolved in borate buffer (pH 8.5) and added to the PEI-FITC solution. Sodium cyanoborohydride (NaBH_3_CN) was added as a selective reducing agent. The mixture was stirred for 72 h at room temperature, facilitating the formation of stable secondary amine linkages between the polymer amines and the open-chain aldehyde forms of the carbohydrates.

**CDI-Mediated Coupling (Ligands L2, L4).** To synthesize ligands with cyclic carbohydrate presentation (ManCyc, L2 and GalCyc, L4), a 1,1′-carbonyldiimidazole (CDI)-mediated coupling reaction was employed to preserve the pyranose ring structure. The carbohydrate was pre-activated with CDI in anhydrous DMSO for 2 h to generate the reactive imidazolyl carbamate intermediate. The PEI-FITC conjugate was then added, and the reaction proceeded for 24 h. This method targets the primary hydroxyl groups of the sugars, forming stable carbamate linkages with the PEI backbone.

The molar ratios of reagents were strictly optimized for each ligand type (see Table 1) to achieve the desired degree of functionalization. The resulting polymer-based ligands were purified by extensive dialysis (MWCO 1000 Da) against deionized water for 48 h to remove unreacted reagents, free carbohydrates, and salts, followed by lyophilization to obtain the final products as yellow-orange powders.

### 4.3. Instrumental Characterization of Carbohydrate-Functionalized Fluorescent Ligands

The chemical structure and purity of the synthesized conjugates were confirmed using spectroscopic methods. Fourier-transform infrared (FTIR) spectra of ligand solutions (10 mg/mL in PBS) were acquired using a Bruker Tensor 27 spectrometer (Bruker, Bremen, Germany) equipped with a liquid nitrogen-cooled mercury cadmium telluride (MCT) detector. Surface analysis of solid-state polymers was performed using a MICRAN-3 FTIR microscope (Simex, Novosibirsk, Russia), also utilizing a liquid nitrogen-cooled MCT detector.

Nuclear magnetic resonance (NMR) spectra of the polymer solutions (20 mg/mL in D_2_O) were recorded on a Bruker DRX-500 spectrometer (Bruker, Bremerhaven, Germany) operating at frequencies of 500.13 MHz for ^1^H and 125.76 MHz for ^13^C. Chemical shifts are reported in parts per million (ppm) and were referenced to the residual solvent signal.

The physicochemical properties of the ligands, including hydrodynamic diameter and surface charge (zeta-potential), were determined by dynamic light scattering (DLS) and electrophoretic light scattering, respectively. Measurements were conducted using a Zetasizer Nano S (Malvern Instruments, Worcestershire, UK) equipped with a 4 mW He–Ne laser (633 nm) at a fixed scattering angle of 173° and a temperature of 25 °C. Experimental data processing and analysis were performed using Zetasizer Software (v. 8.02).

The specific binding affinities of the synthesized ligands were assessed by determining the dissociation constant (*K*_dis_) using a concanavalin A (ConA) binding assay, which serves as a validated model for mannose receptor interactions using a standardized protocol [75]. To evaluate ligand interactions, molecular dynamics (MD) simulations were performed for the CD206 receptor (PDB ID: 7JUE [76]). Simulations were conducted over 100 ns using NAMD with the AMBER ff14SB force field across three replicates [65]. Binding free energies were calculated utilizing the MM-PBSA method, which demonstrated a moderate correlation with experimental FTIR p*K*_d_ values (r = 0.68). Furthermore, Concanavalin A (ConA) was validated as a robust structural model for CD206, exhibiting a high correlation (r > 0.90). To predict ligand affinities across all three target receptors—CD206, CD301 (PDB ID: 6PY1 [77]), and CD209 (PDB ID: 2IT5 [78])—we employed the Pafnucy neural network. This 3D convolutional neural network (3D-CNN), trained on the PDBbind2020 dataset, achieved an overall test accuracy of r = 0.82, and specifically r = 0.80 for CD206. Affinity predictions were generated utilizing voxel grid representations (20 × 20 × 20 Å, with a 0.5 Å resolution), and binding constants were derived via energy normalization according to the following equation *K*_d_ = exp((ΔG + α)/(R·T)).

### 4.4. Patient Sample, Ethical Statement, Clinical Severity Scoring and Prognostic Classification

In this study, we utilized a primary overarching cohort of 72 pediatric patients presenting with distinct chronic and recurrent respiratory pathologies—predominantly including asthma, bronchiectasis, and bronchitis—to develop the baseline predictive LDA model. To demonstrate the platform’s capacity to detect dynamic drug-induced phenotypic shifts, a targeted sub-cohort of 10 patients was selected from this primary group for deep functional profiling and ex vivo pharmacological remodeling. The selection of this subset was deliberately designed to capture diverse baseline macrophage polarization states (ranging from M1-driven to M2a-skewed profiles).

De-identified bronchoalveolar lavage fluid (BALF) samples were prospectively collected at the Clinical Hospital (Moscow, Russia) from ten pediatric patients (aged 6–16 years) presenting with diverse respiratory conditions, including bronchiectasis, bronchial asthma, and acute bronchitis. All procedures complied with the Declaration of Helsinki and were approved by the Local Ethics Committee (Protocol #2024-15A). Informed written consent was secured from the legal guardians of all participants prior to enrollment.

To systematically categorize the diverse inflammatory profiles of the clinical cohort and provide a continuous variable for the downstream Linear Discriminant Analysis (LDA), a standardized Clinical Severity Score was developed. This cumulative index (ranging from 0 to 5) was calculated based on five primary systemic biomarkers: total White Blood Cell (WBC) count, Neutrophil fraction (%), Eosinophil fraction (%), Monocyte fraction (%), and serum C-Reactive Protein (CRP) concentration.

To maximize clinical interpretability and practical utility, an unweighted binary scoring rubric was employed. For each of the five parameters, a score of 1 was assigned if the measured value fell outside the established local laboratory reference intervals (either elevated or depressed), and a score of 0 was assigned if the value was within the normal physiological range. While the presence of fever was recorded as a supplementary clinical observation, it was excluded from the numeric index to maintain a strict focus on hematological and biochemical deviations. Preliminary testing with weighted formulas or continuous degrees of deviation obscured the direct biological interpretability of the score without enhancing class separation. Therefore, this straightforward additive model was utilized to stratify patients into three robust prognostic cohorts: Good prognosis (Severity Score = 0; no systemic deviations), Neutral prognosis (Severity Score = 1–2; mild to moderate systemic involvement), and Poor prognosis (Severity Score ≥ 3; severe, multi-parameter systemic dysregulation).

### 4.5. Processing and In Vitro Remodeling of BALF Cells

Upon collection, BALF samples were immediately transported to the laboratory on ice to preserve cell viability. The fluid was filtered through a sterile 100 µm cell strainer (**Corning^®^ cell strainer, CLS431752**) to remove mucus and particulate debris. The cellular fraction was pelleted by centrifugation at 400× *g* for 10 min at 4 °C. The supernatant was discarded, and the cell pellet was washed twice with cold sterile PBS (pH 7.4).

The resulting cell pellet was resuspended in complete RPMI-1640 culture medium supplemented with 10% heat-inactivated fetal bovine serum (FBS), 2 mM L-glutamine, and a penicillin–streptomycin mixture (100 U/mL penicillin and 100 µg/mL streptomycin). To accurately reflect the native pulmonary microenvironment and prevent the loss of valuable or fragile cellular subpopulations, the unfractionated (whole) BALF cellular suspension was utilized for both the primary diagnostic profiling and the dynamic in vitro pharmacological remodeling assays.

For the phenotypic remodeling assays, the whole BALF cell suspensions were incubated for 24 h in fresh medium containing specific therapeutic agents (Table 3). The rationale for the selected drug concentrations was established through preliminary in vitro dose-finding utilizing standard MTT (3-[4,5-dimethylthiazol-2-yl]-2,5 diphenyl tetrazolium bromide) assays. The primary methodological objective was to identify the maximum final working concentration that strictly maintained high cellular viability (>80%). This stringent viability threshold was critical to ensure that any subsequent changes in ligand-binding profiles reflected true, active functional repolarization rather than artifacts of sublethal cellular stress or selective cytotoxicity.

Based on the MTT viability profiles, the final in vitro concentrations were rigorously stratified. For inherently cytotoxic chemotherapeutics (paclitaxel, vincristine, doxorubicin, and cisplatin), the final exposure concentration was restricted to 2 μg/mL, as higher doses induced significant mortality. For doxycycline, the optimal immunomodulatory, non-toxic final concentration was 25 μg/mL. For the remaining standard modulators and metabolites, the cells safely tolerated a concentration of 100 μg/mL. A comprehensive summary of the specific tested final concentrations and corresponding post-treatment macrophage viability metrics is provided in Table 3.

Conversely, for specific downstream analytical assays requiring highly purified cell populations (flow cytometric quantification), alveolar macrophages were selectively enriched from the BALF suspension using mannan-coated 96-well culture plates (2 h at 37 °C, 5% CO_2_). Non-adherent cells were removed by gentle washing. To mitigate potential artifacts related to transient receptor occupancy induced by the mannan trap, the adhered macrophages underwent a resting incubation period in fresh medium to facilitate receptor recycling prior to testing. This protocol consistently yielded >90% macrophage purity and >85% viability.

Flow cytometric analysis of the enriched macrophages was conducted using a BD FACSAria™ III Cell Sorter (BD Biosciences, San Jose, CA, USA). To precisely quantify the cellular uptake of the FITC-conjugated polymers, a standardized three-gate strategy was applied to the FITC channel: G1 delineated the unbound cell population; G2 delineated cells with moderate baseline surface interaction; and G3 delineated the subpopulation characterized by high-intensity, specific receptor binding.

### 4.6. Immunofluorescence Microscopy of Primary Macrophages and Control Cell Lines

To establish robust biological baselines and rule out non-specific interactions, immortalized human cell lines were used alongside primary patient-derived BALF cells. THP-1 human monocytes and CD206-negative human fibroblasts were obtained from the cell line bank of Lomonosov Moscow State University. THP-1 cells were cultured in RPMI-1640 medium (Gibco, Grand Island, NY, USA) supplemented with GlutaMAX™ Supplement (Gibco), 10 mM HEPES (pH 7.4), 10% heat-inactivated FBS (Gibco, Grand Island, NY, USA), and a 1% antimycotic-antibiotic solution (HyClone, Logan, UT, USA) at 37 °C in a humidified 5% CO_2_ atmosphere. To differentiate the non-adherent THP-1 monocytes into macrophage-like cells, the suspension (0.5 × 10^6^ cells/mL) was stimulated with 100 nM phorbol 12-myristate 13-acetate (PMA, p8139, Sigma-Aldrich) for 72 h. Subsequently, the differentiation medium was replaced with fresh complete medium, and the cells were rested for an additional 96 h to stabilize the macrophage phenotype prior to the assays. Fibroblasts were cultured under standard adherent conditions and served as a definitive negative cellular control.

To visualize specific ligand-receptor colocalization, the target cells (patient-derived BALF macrophages, THP-1-derived macrophages, and negative control fibroblasts) were incubated with the respective FITC-labeled carbohydrate ligands (1 mg/mL) for 2 h. To rigorously confirm the absence of non-specific binding of the polyethylenimine (PEI) scaffold, the negative control fibroblasts were additionally tested at an extreme ligand concentration of 10 mg/mL.

After washing with PBS to remove unbound polymers, the cells were processed for immunofluorescence staining. To prevent non-specific antibody binding, cells were blocked with 10% normal goat serum (Sigma-Aldrich, St. Louis, MO, USA) in PBS containing 1% BSA (PanEco, Moscow Russia) for 1 h at room temperature (RT). The cells were then incubated for 2 h at RT with an anti-CD206 primary antibody (ab64693, Abcam, Cambridge, UK; 1:100) to target the mannose receptor, or a rabbit IgG isotype control (910801, BioLegend, San Diego, CA, USA; 1:100) to assess background staining. Following three consecutive washes with PBS, the samples were incubated with an Alexa Fluor 594-conjugated Goat anti-Rabbit secondary antibody (A11037, Invitrogen, Carlsbad, CA, USA; 1:1000) for 1 h at RT in the dark. Finally, cell nuclei were counterstained with DAPI (1 µg/mL in PBS; Sigma-Aldrich) for 5 min prior to imaging.

### 4.7. Label-Free FTIR Chemical Mapping

BALF macrophages were incubated with the respective carbohydrate ligands (1 mg/mL) for 2 h. Following incubation, the cells were washed twice with buffer to remove any unbound polymer. The cell suspensions were then deposited onto mirrored metal plates and allowed to air-dry completely. Chemical mapping was conducted using a MICRAN-3 FTIR microscope (Simex, Novosibirsk, Russia) equipped with a mercury cadmium telluride (MCT) detector. Spectral data were acquired over a 100 × 100 µm area with a spatial resolution of 2.5 µm, accumulating 30 scans per single point.

### 4.8. Fluorescent Fingerprint Analysis of BALF Samples—Macrophage Phenotyping

The experimental protocol commenced with isolation of cells from BALF, followed by plating the cell suspension into the wells of a microtiter plate to allow cellular adherence. Subsequently, a panel of fluorescent probes was introduced, and the mixture was incubated for 4 h under controlled conditions. Fluorescence measurements were then performed on the reaction mixture, enabling calculation of binding parameters. Using five different fluorescent markers tested both in the presence and absence of mannans allowed generation of ten binding indices per sample, which constitute the basis for our fingerprint profiling approach.

The total reaction volume was maintained at 250 µL, containing approximately 1 × 10^6^ BALF cells. The fluorescent marker was applied at final concentration of 0.05 mg/mL, while mannan as competitor was also used at 0.05 mg/mL. The assays were carried out in phosphate-buffered saline (PBS, 0.01 M, pH 7.4) at 37 °C for 4 h. Fluorescence of FITC-labeled markers was detected using a SpectraMax M5 microplate reader (Molecular Devices Corporation, San Jose, CA, USA) within black-walled Costar 96-well plates featuring clear bottoms. Excitation and emission wavelengths were set at 480 nm and 520 nm, respectively. The binding index, expressed as a percentage, was derived from the difference between initial and final fluorescence intensities, representing the proportion of fluorescent polymer bound to cells.

### 4.9. Reference Macrophage Generation and Phenotypic Deconvolution Analysis

To establish quantitative reference signatures for phenotypic profiling, control macrophage populations were generated from peripheral blood mononuclear cells (PBMCs) obtained from healthy donors. PBMCs were isolated via density gradient centrifugation and differentiated into resting M0 macrophages by culturing in RPMI-1640 supplemented with 50 ng/mL macrophage colony-stimulating factor (M-CSF) for 7 days. Subsequently, M0 macrophages were polarized into the classically activated (M1) phenotype by incubation with 100 ng/mL lipopolysaccharide (LPS) and 20 ng/mL interferon-gamma (IFN-γ), or into the alternatively activated (M2a) phenotype using 20 ng/mL interleukin-4 (IL-4) for 24 h.

The normalized ligand-binding fingerprints (fluorescence metrics across the 5-ligand panel, tested both with and without mannan competition) obtained from these phenotypically distinct reference populations (M0, M1, and M2a) served as the standard basis vectors. To determine the phenotypic composition of the patient samples, the 10-dimensional binding profile of each clinical BALF macrophage sample (S_BALF) was mathematically modeled as a linear combination of these three reference signatures:

S(BALF) = c1 × V(M0) + c2 × V(M1) + c3 × V(M2a) + ε,

where V_i_ represents the 10-dimensional reference vectors, c_i_ represents the unknown fractional coefficients, and ε represents the residual error vector. To solve this system, a Non-Negative Least Squares (NNLS) minimization algorithm was employed [38]. The strict non-negativity constraint (c_i_ ≥ 0) was applied to ensure biological validity, preventing the calculation of negative cellular populations. The algorithm minimized the sum of squared residuals (||ε||^2^) between the experimental BALF vector and the theoretical reconstructed vector. The resulting coefficients (c1, c2, c3) were normalized and converted to percentages, representing the relative fractional contributions of the M0, M1, and M2a subpopulations. The unmapped variance—derived from the magnitude of the error term ε—was quantified as the “Residual” fraction, encompassing atypical or transitional macrophage states not captured by the primary tripartite axis.

To assess the therapeutic efficacy of the tested compounds, a Pearson correlation analysis was performed. The binding profile of each drug-treated sample was correlated against the averaged reference centroids derived from the “Good Prognosis” (Severity Score 0) and “Poor Prognosis” (Severity Score 5) clinical cohorts. A statistically significant positive shift in the correlation coefficient (r) toward the “Good Prognosis” reference profile was interpreted as a successful functional remodeling of the alveolar macrophages.

### 4.10. Computational Framework and Linear Discriminant Analysis (LDA)

To statistically discriminate between clinical prognostic groups based on complex macrophage ligand-binding signatures, a multivariate classification approach using Linear Discriminant Analysis (LDA) was employed. All computational procedures were executed within the Jupyter Lab interactive environment using the Python programming language (version 3.9). The analytical workflow integrated Pandas and NumPy libraries for data structuring, normalization, and pre-processing, while the Scikit-learn library was utilized for the implementation of the LDA algorithm. High-dimensional data visualization and canonical discriminant plotting were performed using Matplotlib (version 3.7.1) and Seaborn (version 0.12.2).

The input feature matrix was constructed using ten quantitative variables per patient sample: the normalized binding indices of the five fluorescent ligands (L1–L5) measured under standard conditions, and the corresponding indices measured in the presence of competitive mannan (ML1–ML5). The clinical prognosis category (“Good”, “Neutral”, “Poor”) served as the target variable.

The LDA algorithm projected the high-dimensional feature space onto a reduced canonical space (LD1 and LD2) optimized to maximize the ratio of between-class variance to within-class variance (Fisher’s criterion). The first canonical discriminant function (LD1) was extracted to quantify the separation between favorable and unfavorable clinical outcomes. Model performance was evaluated using confusion matrices and classification accuracy scores calculated within the computational framework. Decision boundaries were visualized by generating a prediction mesh grid across the 2D canonical space to map the classification regions for each coordinate point.

### 4.11. Statistical Analysis and Predictive Modeling

Quantitative data are presented as the mean ± standard deviation (SD). Statistical methodologies were selected based on the specific experimental design. For cross-sectional comparisons among multiple independent groups, such as the binding affinities of distinct ligand formulations, a one-way analysis of variance (ANOVA) followed by Tukey’s post hoc test was applied. To assess dynamic, within-patient pharmacological shifts (baseline versus post-treatment macrophage profiles), paired Student’s *t*-tests were utilized. Statistical significance was defined as *p* < 0.05.

Linear Discriminant Analysis (LDA) was employed on the multidimensional ligand-binding dataset from the 72-patient cohort to develop a supervised predictive classifier. Model performance and classification accuracy (97.44%) were validated using a Leave-One-Out Cross-Validation (LOOCV) approach at the patient level to prevent overfitting. All statistical and multivariate analyses were performed using the Python scipy.stats module and Origin 2024b statistical software (OriginLab Corporation, Northampton, MA, USA).

## 5. Conclusions

The transition from empirical immunopharmacology to data-driven precision immune engineering requires a fundamental reassessment of how therapeutic interventions are evaluated. To address this challenge, we developed an ex vivo multiparametric test system specifically designed to detect secondary, off-target immunomodulatory effects of pharmacological agents on macrophages. By utilizing patient-derived bronchoalveolar lavage (BAL) samples to interrogate macrophage plasticity via carbohydrate-functionalized fluorescent ligands, the platform generates a highly sensitive, quantitative functional readout of the local immune microenvironment.

Unlike traditional frameworks that restrict macrophages to discrete phenotypic categories, our platform evaluates them across a continuous dynamic spectrum, encompassing pro-inflammatory, anti-inflammatory, and intermediate states. Processing the resulting high-dimensional ligand-binding fingerprints inherently requires artificial intelligence (AI) frameworks. Such Algorithms are highly applicable for such multidimensional data, offering the capacity to identify complex non-linear patterns, predict disease severity prior to clinical manifestation, and stratify patients into distinct prognostic tiers.

We employed Linear Discriminant Analysis (LDA) for the integration of ligand-binding profiles in this study, as it provided a highly efficient, pragmatic, and computationally lightweight solution. LDA achieved an optimal balance between predictive accuracy and data transparency. It enabled the construction of distinct, quantitative immune-state vectors that reliably differentiate favorable prognostic signatures from unfavorable, imbalanced immune states, entirely avoiding the computational overhead and interpretive opacity characteristic of deep neural networks.

In translational terms, quantifying the terminal functional phenotype of macrophages proves more clinically actionable than tracking discrete intracellular drug signaling pathways. LDA modeling allowed us to quantitatively measure the phenotypic shifts in primary patient macrophages toward pro-inflammatory (M1), reparative (M2a), or deactivated (M0) states. Consequently, the assay successfully captured the M2a-polarizing action of doxycycline, the M1-driving effect of 4-methylumbelliferone, and the deactivation to M0-like state induced by paclitaxel.

The clinical validity of these induced phenotypes is strictly governed by the patient’s baseline immune context. Our data confirm that while doxycycline’s robust M2a polarization supports the resolution of acute neutrophilic inflammation, it represents a strict contraindication in Th2-driven diseases such as asthma, where excessive alternative activation exacerbates airway remodeling and fibrosis. The platform also demonstrated the efficacy of targeted natural adjuvants in correcting these adverse secondary pharmacological effects.

Functioning as a comprehensive immune sensitivity assay, the multiparametric test system, integrated with LDA algorithms, establishes a functional framework for personalized companion diagnostics. By centering on the quantitative measurement of terminal macrophage phenotypes, this approach advances the clinical management of severe respiratory pathologies from empirical, trial-and-error treatments to predictive, data-driven immune reconstruction.

To sum up, our study establishes a comprehensive framework for transitioning from empirical immunopharmacology to precision immune engineering. We demonstrate that the efficacy of any therapeutic agent is strictly vector-dependent, dictated by the interaction between the drug’s mechanism and the patient’s unique ligand-receptor landscape. The integrated platform presented here seamlessly addresses three fundamental clinical needs. First, it enables diagnostic stratification by detecting patients with a latent poor prognosis before clinical decompensation occurs. Second, it facilitates personalized screening to filter out agents that, despite general efficacy, would trigger catastrophic off-target polarization in a specific host. Finally, it permits the rational design of combination therapies, pairing primary agents with specific adjuvants to counterbalance therapeutic toxicity and realign the immune vector. The implementation of this predictive navigation system moves the field beyond standardized protocols toward a mathematically rigorous strategy of individualized immune recovery.

## Figures and Tables

**Table 1:** Designation, physicochemical parameters and specificity of carbohydrate-functionalized fluorescent ligands for macrophage receptors. PBS (0.01 M, pH 7.4). T = 37 °C.

Ligand	Designation	Molar Ratio	Hydrodynamic Diameter, nm *	ζ-Potential, mV *	Binding with CD206+ Macrophage, % **	Predicted Affinities for Macrophage Receptors, p*K*_dis_ ***			
						CD206 (Mannose Receptor)	CD209 (DC-SIGN)	CD280 (MRC2/ Endo180)	CD301 (MGL)
**L1**	Man_Lin_-PEI-FITC	15:1:1	105 ± 10	+10 ± 2	38 ± 3	5.2	5.0	4.1	4.0
**L2**	Man_Cyc_-PEI-FITC	18:1:1	115 ± 15		60 ± 7	6.5	5.8	4.5	4.1
**L3**	Gal_Lin_-PEI-FITC	16:1:1	110 ± 15		14 ± 2	4.0	4.1	4.3	5.2
**L4**	Gal_Cyc_-PEI-FITC	13:1:1	115 ± 10		48 ± 4	5.1	5.3	4.0	6.8
**L5**	triMan-GlcNAc_2_-PEI-FITC	10:1:1	130 ± 20		80 ± 6	7.4	6.7	6.1	4.9

* measured by DLS; ** by flow cytometry; *** affinities represent in silico neural-network–based binding predictions using Pafnucy neural network [65,66].

**Table 2:** Complete blood count (CBC) parameters and peripheral inflammatory markers in pediatric patients with respiratory disease. The de-identified cohort (P1–P10) includes age, sex, diagnosis, fever status, total white blood cell (WBC) count, leukocyte differential (neutrophils, eosinophils, and monocytes; %), serum C-reactive protein (CRP), and a clinical disease severity score. Values are reported from venous whole-blood (CBC and differential) and serum (CRP) specimens. Values deviating above or below the standard reference intervals are indicated in bold.

Patient	Age (Years)	Gender	Diagnosis	Fever	WBC, 10^9^/L	Neutrophils (%)	Eosinophils (%)	Monocytes (%)	CRP, mg/L	Severity *	Prognosis
P1	10	M	Bronchiectasis	No	**2.16**	**36.0**	**0.1**	**10.7**	**5.7**	5	Poor
P2	13	M	Bronchial asthma	No	5.45	47.0	4.4	6.0	**12.0**	1	Neutral
P3	7	F	Bronchial asthma	No	**14.13**	42.5	**6.2**	4.0	0.8	2	Neutral
P4	6	M	Bronchiectasis	No	4.96	50.9	1.2	5.8	**6.5**	1	Neutral
P5	11	M	Bronchitis	**Yes**	5.99	50.3	4.6	9.0	**44.7**	3 **	Poor
P6	8	F	Bronchiectasis	No	7.53	53.6	2.3	7.0	2.9	0	Good
P7	16	F	Bronchial asthma	No	**4.33**	58.4	2.0	4.7	0.8	1	Neutral
P8	12	F	Bronchiectasis	No	7.36	43.8	3.4	7.3	1.0	0	Good
P9	14	M	Bronchial asthma	No	**4.34**	46.2	**0.7**	7.0	4.2	2	Neutral
P10	7	F	Bronchial asthma	No	7.87	45.6	4.6	5.9	**10.5**	1	Neutral

* Reference ranges: WBC (4.5–12.0 × 10^9^/L); Neutrophils (37–60%); Eosinophils (1–5%); Monocytes (2–10%); CRP (<5.0 mg/L). Values deviating from these ranges are highlighted in bold. ** Patient P5 was assigned a clinical severity score of 3 and a “Poor” prognosis due to a pronounced acute inflammatory state, evidenced by concurrent fever and a markedly elevated CRP level (44.7 mg/L).

**Table 3:** Comprehensive classification of the tested pharmacological and biologically active agents based on their macrophage remodeling direction. Phenotypic shifts were quantified utilizing least squares deconvolution of patient-derived BALF profiles against established reference M0, M1, and M2a populations. To ensure that observed phenotypic trajectories reflect true active repolarization rather than artifacts of sublethal stress or cytotoxicity, final exposure concentrations were optimized via preliminary MTT dose-finding assays. The table reports the final working in vitro concentrations utilized and the corresponding mean post-treatment alveolar macrophage viability, strictly maintained above an 80% threshold.

Substance Class	Agent	Final Concentration, µg/mL	Viability (MTT, %)	Predominant Effect (Figure 6)	Physiological Interpretation
Antibiotics	Doxycycline 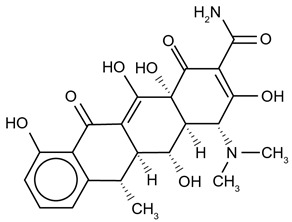	25	91.2 ± 3.5	M2a Polarization (e.g., P7).	High potential for resolving chronic inflammation; however, poses a risk of exacerbating fibrosis in Th2-driven conditions (e.g., asthma).
Cytostatics	Paclitaxel 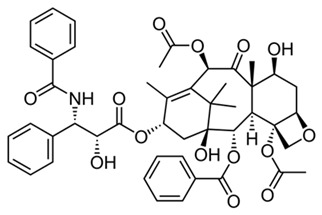	2	82.6 ± 4.1	M0-Shift (Deactivation). Increases marker-negative population.	Disruption of polarization signaling; cellular “reset” or immune tolerance breaking due to cytoskeletal stress.
	Vincristine 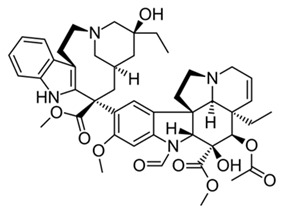	2	84.0 ± 3.8	Slight increase in M1 (e.g., P1).	Pro-inflammatory modulation alongside cytotoxic effects; risks sustaining sterile inflammation.
	Doxorubicin 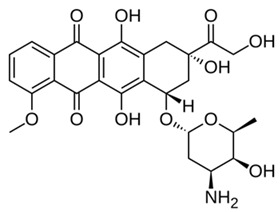	2	83.1 ± 4.4	M1/M0 Mixed. maintains M1.	Induction of immunogenic cell stress and acute pro-inflammatory signaling.
	Cisplatin 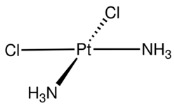	2	81.5 ± 5.0	M1 Maintenance. Maintains high inflammatory profile.	Preservation of the chronic inflammatory state under severe cellular stress.
Pharmaceuticals (Modulators) and Adjuvants	Cyclovalone 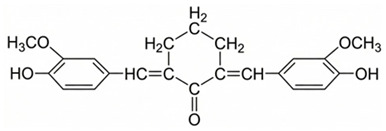	100	93.4 ± 2.9	Moderate M2a induction.	Weak modulator with highly variable individual patient responses.
	MUmb (4-Methylumbelliferone) 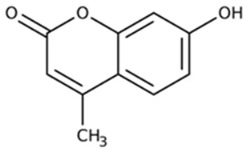	100	89.7 ± 3.2	Induces M1 from M0 (e.g., P3); stabilizes high M1.	Macrophage activation; potential utility as an adjuvant in cancer immunotherapy to heat up “cold” tumors.
	2-mercaptobenzimidazole (2-MBI) 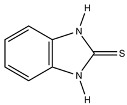	100	87.2 ± 4.5	Strong M1 induction (P5) or M0 shift (P7).	High variability suggests toxicity or stress-response-mediated alternative activation.
	p-anisidine 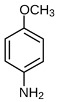	100	88.5 ± 3.7	M2a shift. Reduced M1, increased M2a (P1).	Partial shift towards a tissue-repair phenotype in states of severe inflammation.
	Dillapiol-PPh^3^ 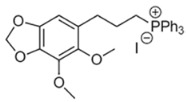	100	90.1 ± 2.8	Moderate M2a shift (P6, P7).	Mild anti-inflammatory activity; potential redox modulation driving resolution.
Nutraceuticals and Metabolites	Curcumin 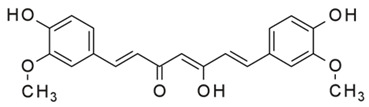	100	92.8 ± 2.4	Context-Dependent. Mainly Decreased M1 (P10).	Adaptogenic effect; outcome depends heavily on the baseline immune status of the patient.
	GSH (Glutathione) 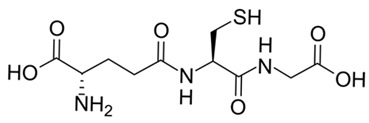	100	95.3 ± 1.8	Redox Support. Decreased M1	Supports cell viability; effect largely mirrors the dominant baseline phenotype.

## Data Availability

The original contributions presented in this study are included in the article and Supplementary Materials. Further inquiries can be directed to the corresponding author.

## Supplementary Materials

The following supporting information [79,80,81,82,83,84,85,86,87,88,89,90,91,92,93,94,95] can be downloaded at: https://www.mdpi.com/article/10.3390/ijms27093894/s1.

## Abbreviations

The following abbreviations are used in this manuscript:

BAL	Bronchoalveolar lavage
BALF	Bronchoalveolar lavage fluid
CPR	C-reactive protein
FITC	Fluorescein isothiocyanate
FTIR	Fourier-transformed infrared spectroscopy
LDA	Linear Discriminant Analysis
