# Supplementary material for: AI-Driven Secondary Immunomodulatory Effects of Conventional Drugs on Patient-Derived Macrophages
Source: Int J Mol Sci. 2026 Apr 27;27(9):3894. doi: 10.3390/ijms27093894 (PMC13163745; doi:10.3390/ijms27093894)
Supplement: Supplementary file 1 [file ijms-27-03894-s001.zip › ijms-4224447-supplementary.pdf]

AI-Driven Secondary Immunomodulatory Effects of Conventional Drugs on Patient-Derived Macrophages

Igor D. Zlotnikov [1,2], Alexander A. Vinogradov [3] and Elena V. Kudryashova [1,2*]

[1] Laboratory of Targeted therapy and Differential Diagnosis, Central University, 7 Gasheka St., Moscow 123056, Russia; izlotnikov2003@yandex.ru
[2] Faculty of Chemistry, Lomonosov Moscow State University, Leninskie Gory, 1/3, 119991 Moscow, Russia
[3] Centre for Mathematical Plasma-Astrophysics, KU Leuven, 3001 Leuven, Belgium; alexander.vinogradov@kuleuven.be
* Correspondence: helenakoudriachova@yandex.ru, el.v.kudryashova@cu.ru

Figure S1. A schematic representation of the synthesis of FITC-labeled fluorescence markers incorporating five different types of carbohydrates that have specific affinities for macrophage receptors, designed to detect them.

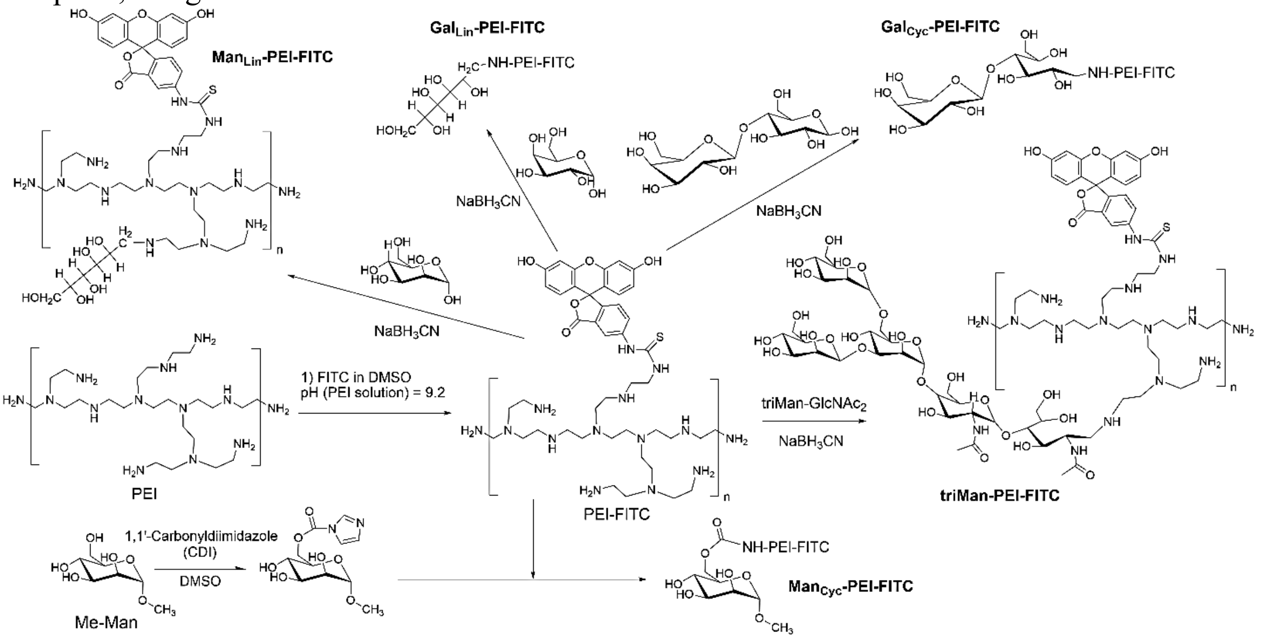

Figure S2. FTIR spectra of FITC ligands with different affinity to CD206, CD301 macrophage receptors X-PEI-FITC. Conditions: PBS (0.01 M, pH 7.4); T = 37 °C.

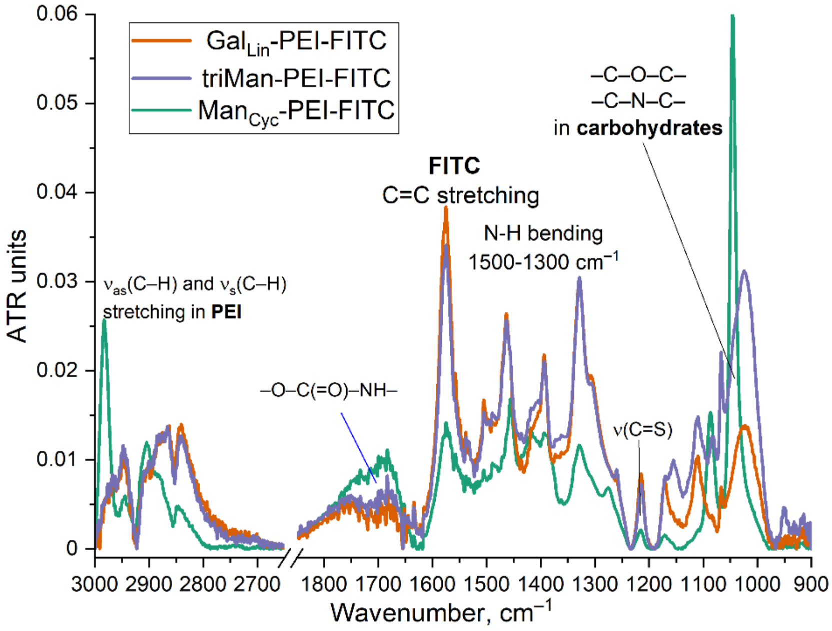

Figure S3. ^{1}H NMR spectra of (a) triMan-GlcNAc2, (b) PEI1.8-triMan with proton assignment. D_2O, 400 MHz.

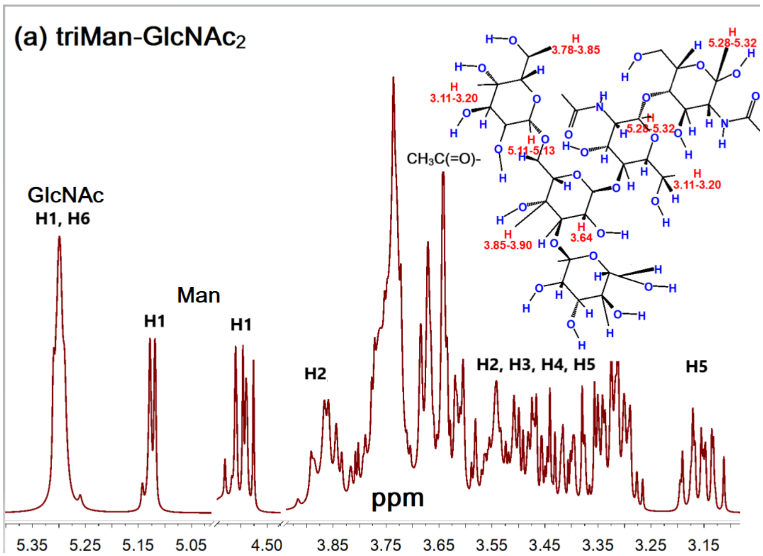

(a)

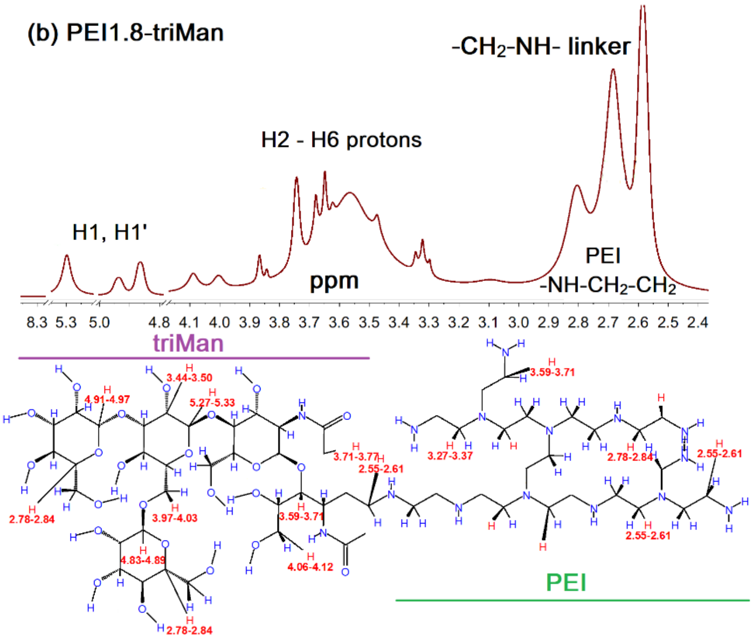

(b)

Figure S4. (a) Flow cytometry results of CD206+ macrophages incubated with X-PEI-FITC polymer samples at concentration of 0.1 mg/mL. (b) Flow cytometry results of human fibroblasts incubated with X-PEI-FITC polymer samples at concentration of 10 mg/mL. Data are presented by overlaid histograms of control macrophages (blue) and macrophages after incubation with sample (red) represented by histograms. (c) Fluorescence microscopy images of human fibroblasts. Cells were stained with anti-CD206 antibodies to visualize the mannose receptor (red, ab64693, Abcam; 1:100), and nuclei were counterstained with DAPI (blue). The carbohydrate-PEI ligands are visualized via their intrinsic FITC label (green). Scale bar = 100 µm.

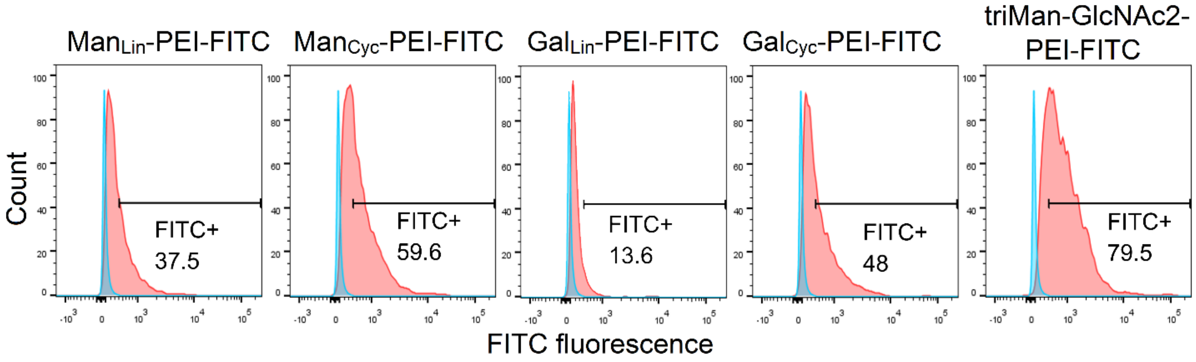

(a)

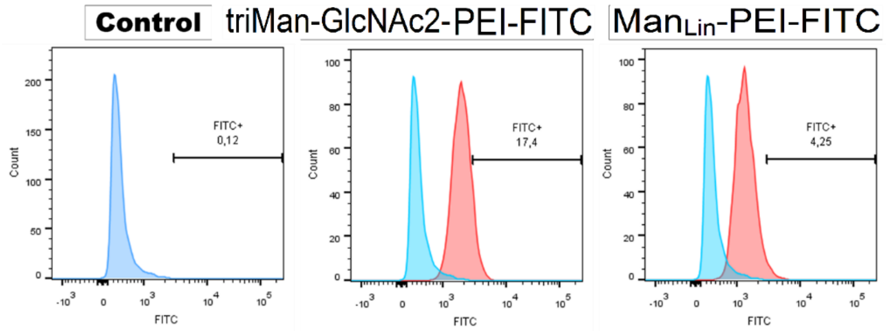

(b)

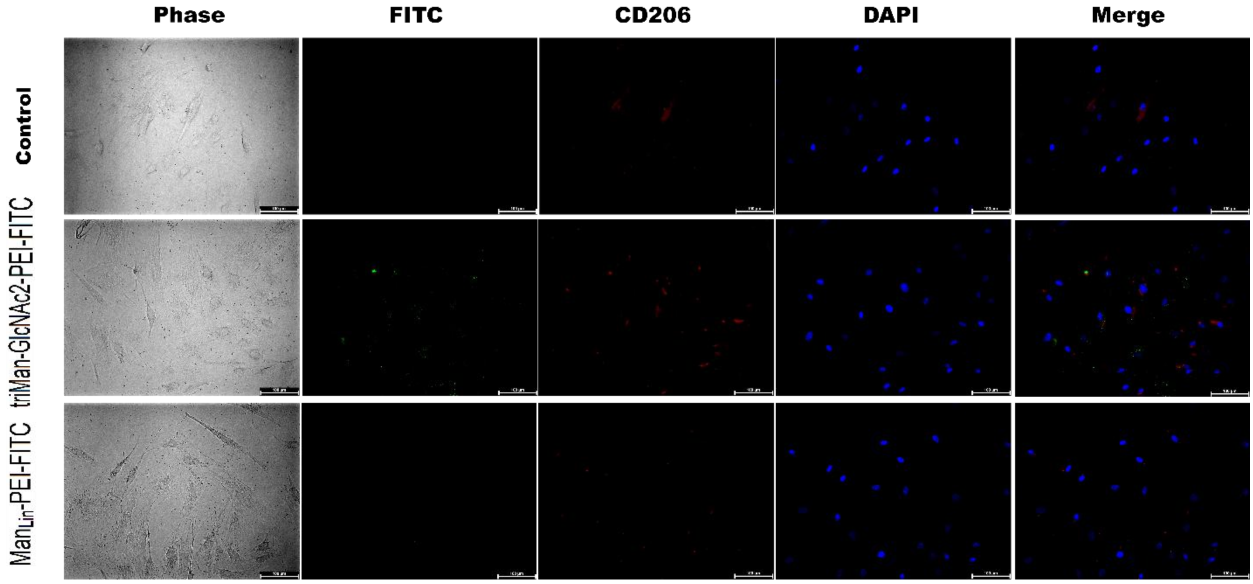

(c)

Figure S5. Feature importance for linear discriminant analysis. Bar chart ranking the top 20 features contributing to the LD1 discriminant function.

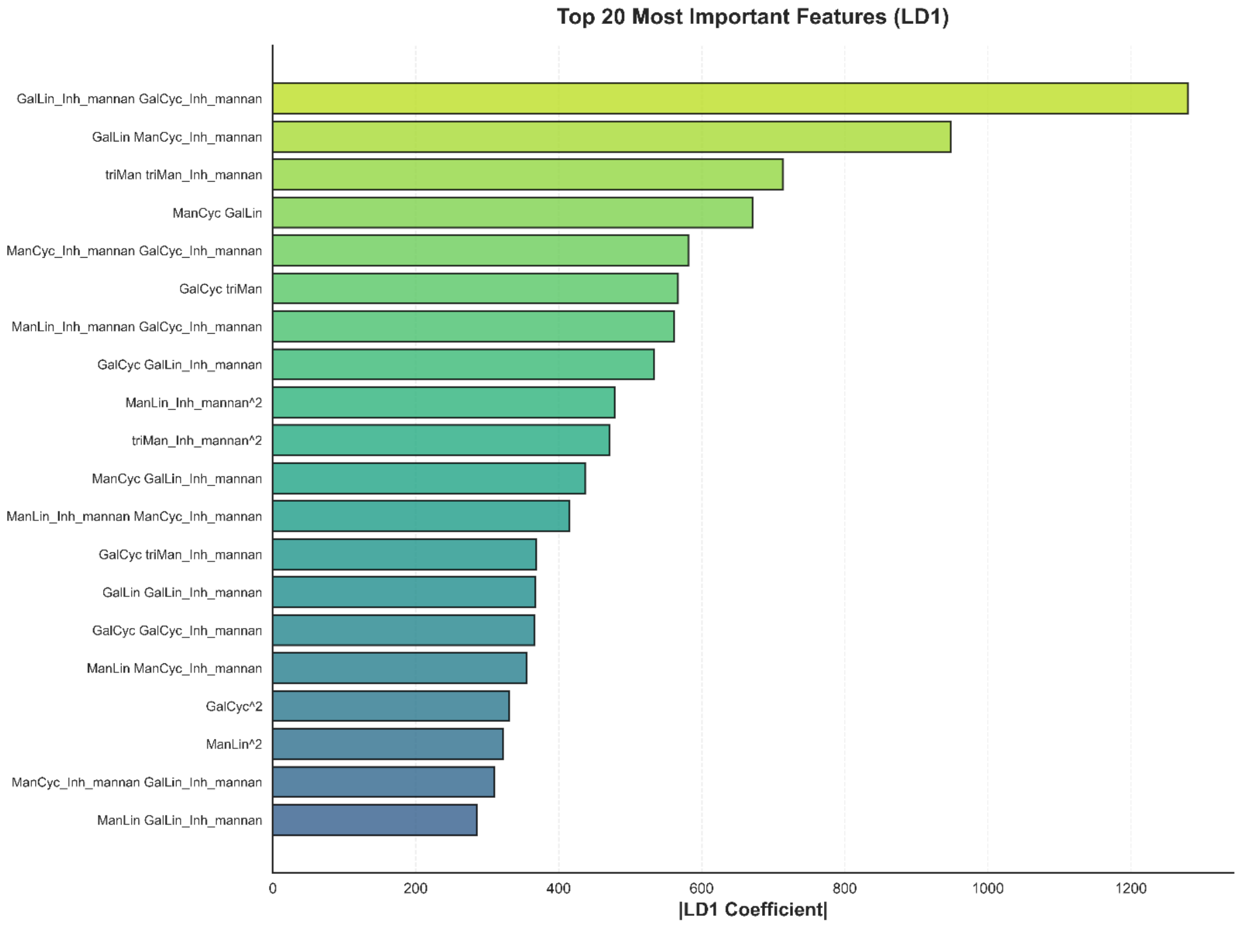

Figure S6. Mannan-mediated inhibition of ligand binding**.** Scatter plots comparing ligand binding in the absence (x-axis) versus presence (y-axis) of mannan inhibition. The diagonal dashed line represents zero inhibition (non-specific binding). 'Good' prognosis samples (green) typically show distinct inhibition patterns (deviation from diagonal), whereas 'Poor' prognosis samples (red) often exhibit aberrant binding behaviors, reflecting the loss of functional receptor specificity identified by the LDA model.

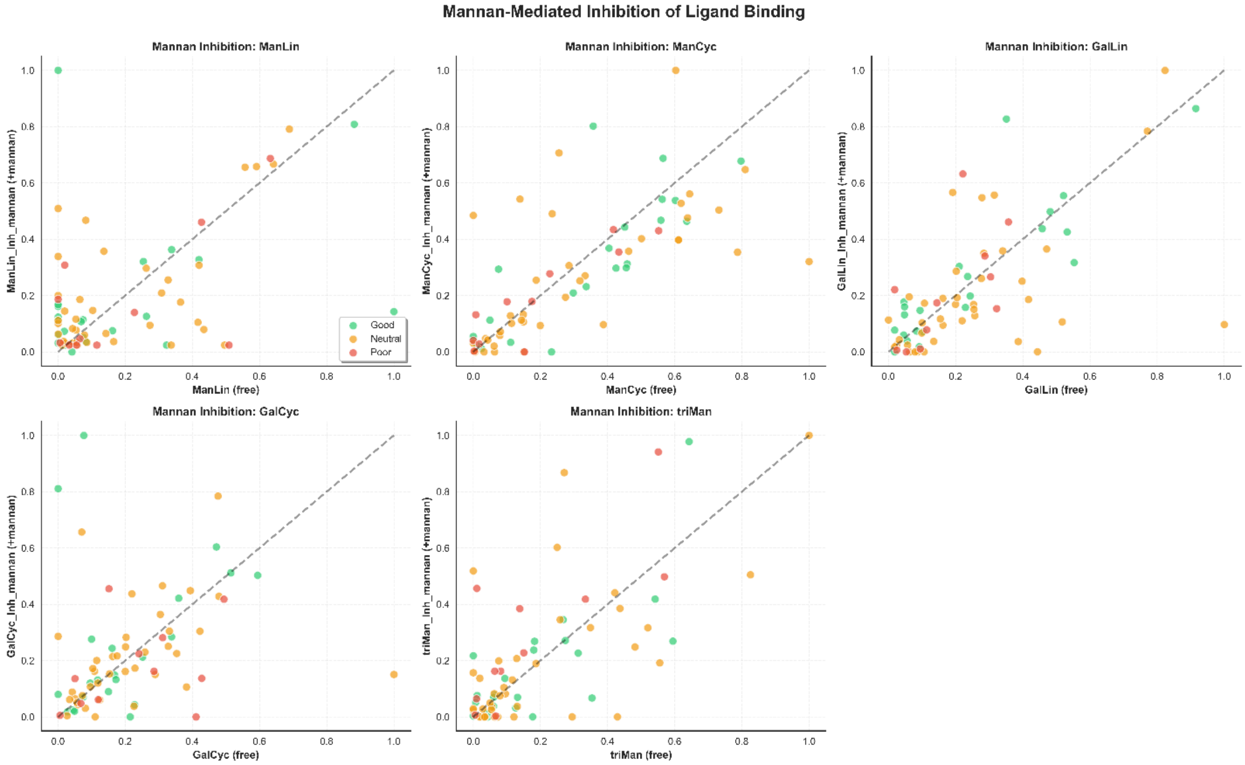

Table S1. LDA-derived contributions of canonical macrophage states in intact and drug-treated BAL samples from pediatric patients. For each patient (P1–P10) and each ex vivo condition (intact BAL and the indicated compounds), the relative contributions (%) of the reference M0-, M1- and M2a-like signatures, together with the residual fraction not captured by these three archetypes, are listed.

Patient	Sample	M0 contribution, %	M1 contribution, %	M2a contribution, %	Residual, %
**P1**	**intact**	**1**	**66**	**1**	**32**
P1	GSH	1	62	7	31
P1	Doxorubicin	1	61	1	37
P1	Vincristine	1	70	2	27
P1	Cisplatin	1	67	4	28
P1	MUmb	1	77	1	21
P1	Doxycycline	51	4	36	9
P1	p-anisidine	6	40	18	37
P1	Paclitaxel	10	65	1	24
**P2**	**intact**	**1**	**55**	**12**	**32**
P2	Paclitaxel	20	56	1	23
P2	GSH	1	70	1	28
P2	MUmb	1	62	1	36
**P3**	**intact**	**44**	**1**	**25**	**30**
P3	Paclitaxel	68	1	14	18
P3	GSH	9	33	41	16
P3	MUmb	1	74	1	24
**P4**	**intact**	**1**	**66**	**1**	**32**
P4	Paclitaxel	11	69	1	19
P4	GSH	1	56	1	42
P4	MUmb	1	67	1	31
**P5**	**intact**	**32**	**28**	**20**	**20**
P5	Paclitaxel	9	32	22	37
P5	dillapiol-PPh3	16	48	1	35
P5	2-mercaptobenzimidazole	1	69	1	29
P5	Curcumin	1	60	1	38
**P6**	**intact**	**1**	**57**	**1**	**41**
P6	Paclitaxel	3	24	41	32
P6	Mumb	1	84	1	14
P6	Cyclovalone	1	68	19	12
P6	dillapiol-PPh3	14	37	40	9
P6	Doxycycline	1	27	49	23
P6	2-mercaptobenzimidazole	32	54	1	13
**P7**	**intact**	**13**	**6**	**38**	**43**
P7	Paclitaxel	43	1	37	19
P7	Mumb	44	1	28	27
P7	Cyclovalone	38	1	38	23
P7	dillapiol-PPh3	38	1	50	11
P7	Doxycycline	1	4	80	15

P7	2-mercaptobenzimidazole	64	14	1	22
**P8**	**intact**	**59**	**18**	**7**	**16**
P8	Doxorubicin	41	35	1	24
P8	Paclitaxel	19	26	15	39
P8	GSH	55	7	26	13
**P9**	**intact**	**1**	**47**	**1**	**51**
P9	Curcumin	1	50	1	48
P9	Cyclovalone	1	51	1	47
P9	Paclitaxel	1	45	1	53
**P10**	**intact**	**1**	**55**	**5**	**39**
P10	Curcumin	17	29	14	39
P10	Cyclovalone	1	59	1	39
P10	Paclitaxel	1	68	1	30

Table S2. Systematic analysis of pharmacological modulators of macrophage polarization: mechanisms and pulmonary applications.

Agent Class	Compound	Primary Molecular Mechanism	Polarization Effect (Literature and Ex Vivo)	Key Markers Modulated	Clinical Application (Lung Context)	Source
**Microtubule Stabilizer**	**Paclitaxel (PTX)**	TLR4 Agonist; MyD88/NF-κB activation	**M2 → M1** (Reprogramming)	↑ iNOS, ↑ TNF-α, ↓ CD206	Reversing fibrosis; Anti-tumor immunity; Breaking M2 tolerance	[1,2]
**Anthracycline**	**Doxorubicin (DOX)**	DNA intercalation; ROS generation	**M2 → M1** (Inflammatory/Toxic)	↑ROS, ↑ iNOS, ↑ Apoptosis	Limited utility due to resident macrophage toxicity	[3]
**Platinum**	**Cisplatin (CDDP)**	DNA crosslinking; Epithelial damage response	**M0 → M1** (Direct); **M1 → M2** (Indirect via Epithelium)	↑ CD86 (early), ↑ TGF-β (late)	Sepsis (bacterial clearance); Potential fibrosis risk	[4,5]
**Tetracycline**	**Doxycycline**	Mitochondrial ribosome inhibition; IL-4R blockade	**Inhibits M2** (Metabolic starvation)	↓ CD206, ↓ Arg1, ↓ MMPs	Asthma remodeling; IPF; Anti-angiogenesis	[6,7]
**Phytochemical**	**Curcumin**	NF-κB inhibition; PPAR-γ activation	**M1 → M2** (Anti-inflammatory)	↓TNF-α, ↓ IL-1β, ↑	Asthma (reducing inflamm	[8,9]

				PPAR-γ	ation); COPD	
**Phenylpropanoid**	**Dillapiol**	CYP450 inhibition; Nrf2 activation	**Anti-inflammatory**; Bio-enhancer	↓IL-6, ↑Nrf2, ↑GSH	Adjuvant therapy; Redox balance	[10]
**Hyaluronan Inhibitor**	**4-Methylumbelliferone**	HAS inhibition (depletes HA coat)	**M2 → M1** (Niche disruption)	↓CD206, ↓TGF-β	Pulmonary Fibrosis; Cancer	[11, 12]
**Benzimidazole**	**2-Mercaptobenzimidazole**	Microtubule/P2Y interaction	**Stimulates Phagocytosis**	↑Phagocytic Index	Bronchiectasis (clearing infection)	[13]
**Synthetic Curcuminoid**	**Cyclovalone**	COX inhibition; Inflammasome block	**Anti-inflammatory**	↓IL-1β, ↓COX-2	Acute exacerbations; Inflammatory resolution	[14]
**Thiol**	**Glutathione (GSH)**	Redox buffer; NF-κB suppression	**M1 → M0** (Resolution)	↓ROS, ↓TNF-α	COPD; Oxidative stress injury	[15]
**Vinca Alkaloid**	**Vincristine**	Microtubule destabilization; NLRP3 activation	**Pro-inflammatory** (Neurotoxic)	↑IL-1β, ↑NLRP3	Neuropathy research (mechanistic control)	[16]
**Toxic Amine**	**p-Anisidine**	Oxidative toxicity; DNA damage	**Toxic / Dysfunctional**	↑ROS, ↓Viability	Toxicology model	[17]

References

1. Wanderley, C.W.; Colón, D.F.; Luiz, J.P.M.; Oliveira, F.F. *et al.* Paclitaxel Reduces Tumor Growth by Reprogramming Tumor-Associated Macrophages to an M1 Profile in a TLR4-Dependent Manner. **Cancer Res.** 2018, **78**, 5891–5900. doi:10.1158/0008-5472.CAN-17-3480
2. Zhu, L.; Chen, L.; Mao, X. *et al.* Low-dose paclitaxel suppresses the induction of M2 macrophages in gastric cancer. **Oncol. Rep.** 2017, **37**, 2178–2186. doi:10.3892/or.2017.5504
3. Zhang, X.; Fu, Y.; Li, H. *et al.* Self-Maintenance of Cardiac Resident Reparative Macrophages Attenuates Doxorubicin-Induced Cardiomyopathy Through the SR-A1-c-Myc Axis. **Circ. Res.** 2020, **127**, 610–627. doi:10.1161/CIRCRESAHA.119.316428
4. Duehrkop, C.; Riehl, A. Low-dose cisplatin administration to septic mice improves bacterial clearance and programs peritoneal macrophage polarization to M1 phenotype. **Pathog. Dis.** 2014, **72**, 111–122. doi:10.1111/2049-632X.12206
5. Geng, H.; Lan, R.; Wang, B. *et al.* M2 macrophage polarization modulates epithelial-mesenchymal transition in cisplatin-induced tubulointerstitial fibrosis. **Lab. Invest.** 2016, **96**, 190–208. doi:10.1038/labinvest.2015.147
6. He, Y.; Yi, M.; Zhang, Y. *et al.* Doxycycline inhibits polarization of macrophages to the proangiogenic M2-type and subsequent neovascularization. **PLoS ONE** 2014, **9**, e89956. doi:10.1371/journal.pone.0089956
7. Qin, Q.; Sun, Y.; Meng, X.; *et al.* Doxycycline repositioning to induce a potent antitumor immune response through macrophage reprogramming. **Biomed. Pharmacother.** 2023, **165**, 115123. doi:10.1016/j.biopha.2023.115123
8. Zhou, Y.; Zhang, T.; Xiao, X. Curcumin Modulates Macrophage Polarization Through the Inhibition of the Toll-Like Receptor 4 Expression and its Signaling Pathways. **Cell. Physiol. Biochem.** 2015, **36**, 631–641. doi:10.1159/000430126
9. Zhang, Y.; Li, X.; Zhang, H. *et al.* Curcumin Reprograms TAMs from a Protumor Phenotype towards an Antitumor Phenotype via Inhibiting MAO-A/STAT6 Pathway. **Cancers** 2022, **14**, 5729. doi:10.3390/cancers14225729
10. Meccia, G.; Mauffette, F.; Legault, J.; Lavoie, S. The anti-inflammatory activity of dillapiole and some semisynthetic analogues. **Molecules** 2011, **16**, 8604–8615. doi:10.3390/molecules16108604
11. Díaz, M.; Granés, F.; Paytubia, W. *et al.* 4-methylumbelliferone-mediated polarization of M1 macrophages correlate with decreased hepatocellular carcinoma aggressiveness in mice. **Oncotarget** 2021, **12**, 567–580. doi:10.18632/oncotarget.27909
12. Yoshida, T.; Kadowaki, K.; Kondo, Y. *et al.* 4-Methylumbelliferone Targets Revealed by Public Data Analysis and Liver Transcriptome Sequencing. **Int. J. Mol. Sci.** 2023, **24**, 2129. doi:10.3390/ijms24032129
13. Zelepuga, E.A.; Shtabrovsky, A.B.; Pchelintseva, N.V. The activating action of mercaptobenzimidazole derivatives on peritoneal macrophages. **Eksp. Klin. Farmakol.** 1991, **54**, 36–38. (https://pubmed.ncbi.nlm.nih.gov/1884800/)
14. MedChemExpress. Cyclovalone Product Information. Available online: https://www.medchemexpress.com/cyclovalone.html (accessed February 6, 2026).
15. Diotallevi, M.; Caporali, S.; Marimpietri, D. *et al.* GSH-C4 Acts as Anti-inflammatory Drug in Different Models of Canonical and Cell Autonomous Inflammation Through NFκB Inhibition. **Front. Immunol.** 2019, **10**, 155. doi:10.3389/fimmu.2019.00155
16. Starobova, H.; Mueller, A.; Goldstein, R. *et al.* Vincristine-induced peripheral neuropathy is driven by canonical NLRP3 activation and IL-1β release. **J. Exp. Med.** 2021, **218**, e20201452. doi:10.1084/jem.20201452
17. Laskin, D.L. Macrophages and inflammatory mediators in chemical toxicity: a battle of forces. **Chem. Res. Toxicol.** 2009, **22**, 1376–1385. doi:10.1021/tx900060w
